# Exercise interventions for people diagnosed with cancer: a systematic review of implementation outcomes

**DOI:** 10.1186/s12885-021-08196-7

**Published:** 2021-05-30

**Authors:** Louise Czosnek, Justin Richards, Eva Zopf, Prue Cormie, Simon Rosenbaum, Nicole M. Rankin

**Affiliations:** 1 Mary MacKillop Institute for Health Research, Australian Catholic University, Melbourne, Victoria 3000 Australia; 2 Faculty of Health, Victoria University of Wellington, Wellington, New Zealand; 3 School of Public Health, Faculty of Medicine and Health, University of Sydney, Sydney, Australia; 4 Peter MacCallum Cancer Centre, Melbourne, Victoria 3000 Australia; 5 Sir Peter MacCallum Department of Oncology, The University of Melbourne, Melbourne, Victoria 3010 Australia; 6 School of Psychiatry, University of New South Wales, Sydney, Australia; 7 Black Dog Institute, University of New South Wales, Sydney, Australia

**Keywords:** Exercise, Implementation outcomes, Cancer, Physical activity, Systematic review

## Abstract

**Purpose:**

Exercise is efficacious for people living after a cancer diagnosis. However, implementation of exercise interventions in real-world settings is challenging. Implementation outcomes are defined as ‘the effects of deliberate and purposive actions to implement new treatments, practices, and services’. Measuring implementation outcomes is a practical way of evaluating implementation success. This systematic review explores the implementation outcomes of exercise interventions evaluated under real-world conditions for cancer care.

**Methods:**

Using PRISMA guidelines, an electronic database search of Medline, PsycInfo, CINAHL, Web of Science, SportsDiscus, Scopus and Cochrane Central Registry of Controlled Trials was conducted for studies published between January 2000 and February 2020. The *Moving through Cancer* registry was hand searched. The Implementation Outcomes Framework guided data extraction. Inclusion criteria were adult populations with a cancer diagnosis. Efficacy studies were excluded.

**Results:**

Thirty-seven articles that described 31 unique programs met the inclusion criteria. Implementation outcomes commonly evaluated were *feasibility* (unique programs *n* = 17, 54.8%) and *adoption* (unique programs *n* = 14, 45.2%). Interventions were typically delivered in the community (unique programs n = 17, 58.6%), in groups (unique programs *n* = 14, 48.3%) and supervised by a qualified health professional (unique programs *n* = 14, 48.3%). Implementation outcomes infrequently evaluated were *penetration* (unique programs n = 1, 3.2%) and *sustainability* (unique programs n = 1, 3.2%).

**Conclusions:**

Exercise studies need to measure and evaluate implementation outcomes under real-world conditions. Robust measurement and reporting of implementation outcomes can help to identify what strategies are essential for successful implementation of exercise interventions.

**Implications for cancer survivors:**

Understanding how exercise interventions can be successful implemented is important so that people living after a cancer diagnosis can derive the benefits of exercise.

**Supplementary Information:**

The online version contains supplementary material available at 10.1186/s12885-021-08196-7.

## Background

Cancer is a leading cause of disease burden worldwide. In 2020, 19.2 million new cases of cancer and 9.9 million cancer-related deaths occurred globally [1]. Cancer rates are projected to rise steadily in the coming decades, in part due to population growth, ageing and more people surviving a cancer diagnosis because of improvements in early detection and treatment advances [2, 3].

Exercise is important in addressing the sequala of disease and impacts of a cancer diagnosis, as demonstrated in the robust efficacy base of systematic reviews, meta-analyses and meta reviews [4–11]. High quality or ‘level one evidence’, as gathered through systematic reviews and meta-analyses, informs the development of clinical practice guidelines (CPGs). CPGs are evidence-based statements that include recommendations to optimise patient care [12]. In 2019, the American College of Sports Medicine (ACSM) updated evidence-based advice for cancer and exercise testing, prescription and delivery in cancer survivors. The consensus statement provides exercise prescription recommendations for common cancer-related health outcomes including depression, fatigue and quality of life [13]. The ACSM is one of many organisations worldwide that recommend exercise be incorporated within the routine care for people with cancer [14–17].

The development of CPGs, whilst fundamental to informing evidence-based care, is unlikely to directly change clinical practice [18]. To facilitate the implementation of their consensus statement, ACSM published additional resources describing *how* implementation can be fostered [19] and created the *Moving through Cancer* registry to connect people with cancer to local exercise services [20]. This signifies greater attention to translating research findings into practice and moving beyond demonstrating exercise efficacy for different cancer types.

Most research that establishes the efficacy of health interventions is conducted in tightly controlled research settings, focusing on internal validity [21, 22]. Efficacy studies exclude many participants in an attempt to recruit a homogenous sample. Such research studies are often well funded and have access to the required resources needed to deliver the evidence-based intervention, health program or innovation (hereafter ‘intervention’) with high fidelity to the described study protocol. Further, research staff often take part in extensive training sessions to deliver the intervention [23, 24]. These conditions rarely reflect the conditions under which an intervention is implemented in healthcare settings. That is, staff may have limited time to instruct patients during clinical consultations, inadequate training to prescribe exercise interventions or insufficient physical space to establish an exercise intervention [25]. It is common for efficacious interventions to fail in practice [26] or have reduced clinical impact when replicated to reach more of the population for which they are intended [27, 28]. Pragmatic study designs seek to address these issues through answering the question “Does this intervention work under usual conditions?” [29]. That is, they seek to reflect population diversity in study samples and explore whether it is realistic to implement the intervention. Despite the growth in cancer studies about exercise in recent years, relatively little is known about the outcomes of exercise interventions when implemented using pragmatic study designs, or the ‘external validity’ of how best to implement and evaluate exercise interventions in practice [22].

Proctor and colleagues [30] have developed an Implementation Outcomes Framework to evaluate implementation success. If implementation is successful, the proposed theory of change suggests this contributes to desired clinical or health service outcomes (e.g., a safe, efficient service that successfully addresses patient symptomology). Evaluating the outcomes of implementation efforts can also reduce the risk of incorrectly concluding that an intervention is ineffective, when in fact, poor implementation may be the most significant contributor to failure [30, 31]. Implementation science frameworks that evaluate implementation outcomes may therefore be useful to determine whether failure is due to the intervention or the implementation process [32, 33]. Proctor and colleagues [30] define eight implementation outcomes for this purpose: acceptability, adoption, appropriateness, cost, feasibility, fidelity, penetration and sustainability.

The Implementation Outcomes Framework was used to inform the outcomes of interest for this review. The aim of this review was to examine the implementation outcomes that are evaluated under real-world conditions when exercise interventions are implemented for the care of people diagnosed with cancer.

Table 1 provides a description of how the implementation outcomes were operationalised in this study.

**Table 1:** Operational definition of implementation outcomes applied in review

Implementation outcome	Proctor et al. definitions of outcomes [29]	Operational definition as applied in this review
Acceptability	The perception among implementation stakeholders that a given treatment, service, practice, or innovation is agreeable, palatable, or satisfactory.	The degree to which the patient or healthcare workforce find the exercise intervention satisfactory as measured by the patient or healthcare workforce.
Adoption	The intention, initial decision, or action to try or employ an innovation or evidence-based practice	Any measure that reports on the uptake of exercise intervention as reported by the healthcare staff (for example, total number of staff making referrals to exercise) or organisation; this may include barriers and enablers.
Appropriateness	The perceived fit, relevance, or compatibility of the innovation or evidence-based practice for a given practice setting, provider, or consumer; and/or perceived fit of the innovation to address a particular issue or problem.	Exercise interventions are implemented because there is a specific, documented rationale that indicates the intervention is relevant to that patient population, based on clinical trials effectiveness (for example, reference to a successful efficacy trial that the current exercise intervention is based upon).
Cost	Cost (incremental or implementation cost) The cost impact of an implementation effort according to three components: i) cost of delivering the intervention, ii) cost of the specific implementation strategy and iii) the delivery cost according to the setting	The documented cost of implementing the exercise intervention in healthcare settings. This includes costs incurred by healthcare organisations such as human and physical/practical resources, or costs associated with use of the intervention.
Feasibility	The extent to which a new treatment, or an innovation, can be successfully used or carried out within a given agency or setting	Intervention attendance and/or attrition rates for the program.
Fidelity	The degree to which an intervention was implemented as it was prescribed in the original protocol or as it was intended by the program developers	The exercise intervention is delivered as described in the documented pre-implementation plan or intervention protocol; if adaptations (tailoring) are required, these are reported either qualitatively or quantitatively.
Penetration	The integration of a practice within a service setting and its subsystems	Patients referred to the intervention reported with consideration to total eligible patient population (for example intervention reach data).
Sustainability	The extent to which a newly implemented treatment is maintained or institutionalized within a service setting’s ongoing, stable operations	Documented evidence that the exercise intervention has been integrated within normal organisational operations (for example, reference to polices, hiring staff, documented care pathways) **and** the long-term (> 12 months) health outcomes of the exercise intervention on adverse treatment-related side effects (such as fatigue, quality of life, physical function and/or symptoms of depression). Whilst Proctor and colleague’s definition of sustainability does not include a measure of clinical effect, it is added as a secondary outcome in this review. This decision was made to confirm that the exercise intervention continues to deliver the intended health benefits that it was implemented to address.

## Methods

### Protocol and registration

This review was registered in the PROSPERO database (CRD42019123791) and conducted in accordance with the Preferred Reporting Items for Systematic Reviews and Meta-Analyses (PRISMA) statement [34].

The search strategy was developed in consultation with a librarian experienced in systematic reviews. First, the search strategy of a recent meta-review that summarised the efficacy of exercise and cancer was replicated and augmented with additional search terms for exercise (e.g., physical activity) [5]. Second, this search was combined with terms derived from the Implementation Outcomes Framework (e.g., adoption, acceptability) [35]. Finally, the reference list of relevant articles and the *Moving through Cancer* program registry were also screened to identify potentially relevant studies [20, 36, 37]. The *Moving through Cancer* registry website was selected for screening because it provides a comprehensive and publicly accessible database that details established exercise interventions for people diagnosed with cancer and supports the implementation of the ACSM recommendations. Details of the search strategy are provided in Supplementary Table 1.

An electronic database search was conducted from January 2000 to 6 February 2020 (Medline, PsychInfo, CINAHL, Web of Science, SPORTDiscus, Scopus and Cochrane Central Register of Controlled Trials). Two reviewers (LC, JR) independently completed the title and abstract screening and full text review. Disagreements were resolved through discussion until a consensus was reached. Where agreement was unable to be reached, a third reviewer was available to inform the final decision (EZ). Covidence software was used to manage the screening and data extraction process [38].

### Definition of terms

Physical activity is defined as “any bodily movement produced by skeletal muscles that requires energy expenditure” [39]. Exercise is “a subset of physical activity that is planned, structured, and repetitive and has as a final or an intermediate objective the improvement or maintenance of physical fitness” [39].

### Inclusion and exclusion criteria

The inclusion and exclusion criteria for this review are summarised in Table 2. All types of physical activity and/or exercise (for example, aerobic, resistance, yoga, tai chi, Pilates, high intensity interval training) were included in the review. There were no restrictions placed on moderators of exercise (for example, supervised and unsupervised, home-based, and community/hospital-based settings, group and individual classes, face-to-face and virtual [online/video]). Further, any studies at translational stages prior to and including efficacy studies were excluded. As such, studies described as effectiveness or implementation/dissemination were included. Definitions for the categorisation of studies is supplied in Supplementary Table 2.

**Table 2:** Inclusion and exclusion criteria for the systematic review

**Inclusion criteria**	
• Studies where an exercise intervention was offered alongside cancer care within the continuum from diagnosis to treatment with curative intent and through to survivorship	
• Studies that included people aged 18 years or older with a confirmed diagnosis of cancer	
• Studies that reported at least one implementation outcome, as per the operational definition	
**Exclusion criteria**	
• Non-human studies	
• Studies not published in English	
• Efficacy trials (defined according to an established classification) [40] (refer to supplementary Table 2 for expanded definitions and categorisations applied in this review)	
• Studies involving patients undergoing end-of-life care (for example, palliative care)	
• Studies involving exercise interventions designed to prevent or reduce the risk of developing cancer	
• Intervention studies where exercise interventions were included within a broader healthy lifestyle program and the independent effects of exercise could not be extracted	
• Studies that did not describe an active intervention	
• Studies that describe the methodological development or testing of an instrument to measure efficacy of an exercise intervention	

### Data extraction and quality assessment

A data extraction tool was developed with reference to the published literature [41]. One author (LC) extracted data on: study type (effectiveness or implementation/dissemination study), implementation outcome, the level at which the implementation outcome was measured (patient, provider, intervention, organisation or a combination) and the exercise intervention composition and setting [19]. The Consensus on Exercise Reporting Template (CERT template) provides reporting recommendations and was used to detail the composition of exercise interventions [42].

Study quality was assessed using one of two tools. The Joanna Briggs Institute (JBI) suite of Critical Appraisal Tools were used to assess quality in quantitative and qualitative studies (the relevant JBI tool was selected for each study based upon the study design) [43]. The Mixed Methods Appraisal Tool was used to critically appraise studies that described a mixed method design [44]. The outcomes of the quality assessment are provided in Supplementary Table 3. An independent compliance check of data extraction and quality assessment was completed by two authors (NR, EZ) for 10% of the included studies.

### Data synthesis and analysis

The Implementation Outcomes Framework guided the initial data synthesis [30]. Data were extracted, collated and analysed based upon the eight implementation outcomes. Quantitative and qualitative results were extracted and analysed concurrently and integrated to produce the final synthesis. Descriptive statistics and frequencies (using the total possible number of outcomes as the denominator) were calculated to synthesise the study type and the total number of implementation outcomes explored in the included studies.

## Results

### Search results

**Fig. 1:**
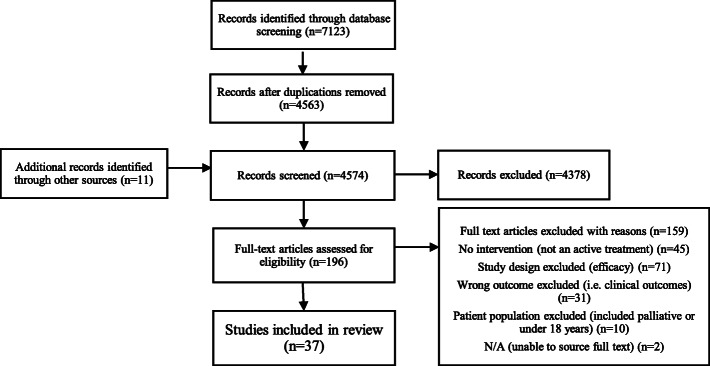
PRISMA Diagram

A total of 7123 articles were identified through the database search. After de-duplication, 4563 articles remained and 11 additional citations were identified through the manual search of reference lists and the *Moving through Cancer* exercise program registry [45–55]. After full text screening, 37 articles were included in the final review, which represented 31 unique programs. Descriptive statistics reported within the manuscript reflect outcomes for unique programs. Figure 1 presents a flow diagram for the results. Supplementary Table 4 provides a list of studies that were excluded after full text review and reasons for exclusion.

Table 3 provides a summary of the characteristics of the studies that met the inclusion criteria.

**Table 3:** Characteristics of included studies

First Author	Year	Study type	Sample size	Implementation Outcome	Level of Analysis	Healthcare setting	Cancer diagnosis	Exercise intervention
Beidas	2014	Effectiveness/ Implementation	*n* = 84 (effectiveness) *n* = 19 (implementation)	Adoption	Healthcare Provider	Community (specialist exercise clinic) + Home	Breast cancer	What (materials) - Power blocks adjustable dumbbells Who (qualifications) – Physiotherapist How (delivery) - One physiotherapist to 7 or fewer survivors; Exercise logs used for self-reported adherence monitoring When, how much (dosage) - 4 small group PT sessions completed over 1–2 months + 2x/wk. home resistance training Tailoring – Individualized
				Appropriateness	Intervention			
				Cost	Intervention			
Bjerre	2018	Effectiveness	*n* = 214 (*n* = 109 intervention *n* = 105 control)	Appropriateness	Intervention	Community (fitness centre)	Prostate cancer	What (materials) – Not reported Who (qualifications) – Local football coaches who underwent 8–10 h of training in intervention and cancer How (delivery) – Group football training When, how much (dosage) - 6 months of recreational football for 1 h 2x/wk. Football sessions lasted 1 h and included 20 min of warm up and 20 min each of drills and match play
				Cost	Intervention			
				Feasibility	Patient			
				Fidelity	Healthcare provider			
Bjerre	2019	Effectiveness	n = 214 (n = 109 intervention n = 105 control)	Appropriateness	Intervention	Community (fitness centre)	Prostate cancer	See Bjerre 2018
Bultijnck	2018	Implementation	*n* = 98	Adoption	Organisation	Hospital (not stated if inpatient or outpatient)	Prostate cancer	Characteristics of exercise program were reported for general cancer rehabilitation programs and prostate cancer specific programs. Below is a summary of both program types: General What (materials) – NA Who (qualifications) - NA How (delivery) - 73.3% were group training, 57.8% started after treatment When, how much (dosage) - Most programs included aerobic and resistance components of between 60 and 90 min duration conducted 2x/wk. 42.2% of programs conducted 24 sessions. Tailoring - NA Prostate cancer specific programs What (materials) – NA Who (qualifications) - NA How (delivery) - 77.8% were group training, 38.9% started during treatment When, how much (dosage) - 100% of programs included aerobic and resistance components and approx. Half also included flexibility and pelvic training, 50% were 90 min in duration and most commonly conducted 2x/wk. 61.1% of programs conducted 48 sessions. Tailoring - NA
Brown	2019	Effectiveness	*n* = 183	Feasibility	Patient	Community (specialist exercise clinic)	Any cancer type	What (materials) - Treadmill, cycle ergometer, NuStep, Aquaciser (underwater treadmill), and outdoor walking or jogging. Cybex® resistance machines, resistance bands, dumbbells, medicine balls, body weight, and resistance tubing Who (qualifications) – Certified Clinical Cancer Exercise Specialists (CCES) How (delivery) - Individual sessions When, how much (dosage) - Each phase (a total of 3 phases + 1 phase of infinite duration) was 3x/wk. for 12 wks. Duration was 60 min per session (20 min aerobic, 30 min resistance (3 × 10 reps), 10 min for flexibility training, and balance exercises incorporated throughout. Intensity increased from low/mod to high as Phases progressed. Tailoring - Individualized based on patient assessment
				Fidelity	Healthcare provider			
Cheifetz	2014	Effectiveness/ Implementation	*n* = 115 (effectiveness)	Fidelity	Healthcare Provider	Community (fitness centre)	Any cancer type	What (materials) -Not reported Who (qualifications) - YMCA staff who undergo specific training lead by physiotherapist or nurse How (delivery)- Group training, peer support encouraged When, how much (dosage) - 12 wk. program, 2x/wk. supervised + 1x/wk. independent exercise. Includes aerobic (target HR 50–80% MHR), muscle strength (2–3 sets, 12 repetitions) and flexibility based on established guidelines (i.e. ACSM) Tailoring - Programs are tailored and individualized on the basis of baseline testing, unique cancer type and stage and person specific precautions and contraindications.
				Feasibility	Patient			
Cheifetz	2015	Effectiveness/ Implementation	*n* = 57 (effectiveness) *n* = 12 (implementation)	Feasibility	Patient	Community (fitness centre)	Any cancer type	See Cheifetz 2014
Culos-Reed	2018	Effectiveness/Implementation	*n* = 58 (effectiveness)	Appropriateness	Intervention	Community (specialist exercise clinic and fitness centre)	Prostate cancer	What (materials) -Exercise that can be completed with minimal equipment (exercise bands and balls, body weights and free weights) Who (qualifications) - Credentialed health and fitness professionals How (delivery)- Group training. Maximum ratio 1 facilitator per 15 participants. When, how much (dosage) - 12-wk program (with an additional 12-wk maintenance phase), 60 min per session, completed 2x/wk. of 1-h duration. A combination of mild to mod/somewhat hard-intensity aerobic and resistance training, or gentle yoga with cooldown and meditation (i.e. savasana). Group exercise consists of 3–4 exercises in a circuit with adapted plyometric aerobic exercise. Tailoring - All exercises are adapted to accommodate individuals’ preferences and limitations
				Cost	Intervention			
				Feasibility	Patient			
				Fidelity	Healthcare Provider			
Culos-Reed	2019	Implementation	n = 11	Cost	Intervention	Community (specialist exercise clinic and fitness centre)	Prostate cancer	See Culos-Reed 2018
Dalzell	2017	Implementation	*n* = 1635 (referred over the duration)	Adoption Cost	Organisation Organisation	Hospital (out-patient) + Community (specialist exercise clinic) + Home	Any cancer type	What (materials) - Not reported Who (qualifications) - Varied depending upon triage of patients How (delivery)- Varied depending upon triage of patients When, how much (dosage) - ACSM exercise guidelines for cancer survivors and included components of flexibility, cardiovascular, and resistance training whenever possible. Focused on increasing physical activity levels and included a combination of home exercise, wellness centre–based training, or participation in exercise classes. Tailoring – Individualized programs with re-assessment every 3 months.
Dennett	2017	Implementation	*n* = 31 (exercise oncology programs) n = 15 (providers)	Adoption	Healthcare Provider	Hospital (in and outpatient) + Community (specialist exercise clinic)	Not reported	What (materials) - Not reported Who (qualifications) - Supervised (by physiotherapists or exercise physiologists) How (delivery) - Typically conducted in a group based upon an initial individualized assessment. When, how much (dosage) - Included aerobic, resistance and flexibility exercises. Exercise outside the program was encouraged with most suggesting aerobic exercise 4-5x/wk. and resistance exercise 2-3x/wk. Strategies used to encourage compliance with home exercise included written home exercise programs and referrals to community groups. Tailoring - Programs are typically individualized, monitored throughout and progressed.
Dolan	2018	Effectiveness	*n* = 152 (files)	Appropriateness	Intervention	Community (specialist exercise clinic)	Breast cancer	What (materials) - free weights, body weight, and/or elastic bands Who (qualifications) - cardiac rehabilitation supervisor + 2 exercise assistants How (delivery)- Group program (15 patients) When, how much (dosage) - supervised exercise (dynamic warmup, aerobic training (walking commence at 1 mile and increase to 3 miles (walk/jog) starting at 60%VO_2_ reserve with fitter individuals starting at 80%VO_2_ reserve), strength training (2 × 10 reps of 12 full-body exercises), and cool-down) with 12 education seminars. 22 sessions 1x/wk. for the duration of the program. In addition to the weekly supervised exercise session, unsupervised activities (up to 2 strength and 4 aerobic sessions /wk) were promoted through education and goal setting. Peer mentoring supported Tailoring - Individualized according to current guidelines and initial baseline fitness test results
				Feasibility	Patient			
Haas	2011	Implementation	NA	Adoption	Organisation	Community (fitness centre)	Any cancer type	What (materials) - Dumbbell weights Who (qualifications) - unclear - clinical personnel complete initial assessment How (delivery) - Unclear When, how much (dosage) - Aerobic exercise, stretching, upper body weight-lifting exercises + 1 core exercise (squats or stability ball). Activity plan developed and a follow-up exercise schedule established. Participants are encouraged to exercise at least 3x/wk. and increase exercise intensity or duration by 10 to 15% each wk. Tailoring - Individualized according to current guidelines and initial baseline fitness test results. Activity is ceased during sessions on self-reported mild fatigue
				Cost	Organisation			
				Feasibility	Patient			
Haas	2012	Effectiveness	*n* = 177	Feasibility	Patient	Community (fitness centre)	Any cancer type	See Haas 2011 Pedometers were provided
				Sustainability	Patient + Organisation			
Heston	2015	Implementation	*n* = 1591 (providers) *n* = 1668 (participants)	Adoption	Organisation	Community (fitness centre)	Not reported	What (materials) - Not reported Who (qualifications) - YMCA staff trained in LIVESTRONG How (delivery)- small-group (6–16 participants) When, how much (dosage) - Adheres to ACSM guidelines. 12-wk duration, 2 sessions/wk. (75 min session) including aerobic fitness, muscle mass and strength, flexibility and balance. Peer-to-peer support included. Tailoring - Instructors created individualized physical activity plan
				Cost	Intervention + Organisation			
				Feasibility	Patient			
				Fidelity	Healthcare Provider			
Irwin	2017	Effectiveness	*n* = 186 (*n* = 95 intervention *n* = 91 control)	Adoption	Organisation	Community (fitness centre)	Any cancer type	See Heston 2015
				Feasibility	Patient			
Kimmel	2014	Implementation	NA	Adoption	Organisation	Community (fitness centre)	Any cancer type	See Haas 2011 After a few months move to 6 to 10 participants per staff member.
				Feasibility	Patient			
Kirkham	2016	Effectiveness	n = 163	Cost	Intervention	Hospital (out-patient)	Any cancer type	What (materials) - Nautilus system Who (qualifications) - 2 exercise physiologists + other health professional How (delivery)- Group-based When, how much (dosage) - 2 x/wk. for 12 wks (24 sessions) with optional education class 1 days per wk. Classes were 60 min in duration and included 20–30 min of aerobic exercise (intensity individualized but between 40 and 80% HHR) + 15–20 min of resistance exercise (8–10 reps increasing to 3 sets before increasing weights, exercise included bicep curl, triceps extension, vertical press, chest press, rows, leg extension, leg curl, leg press, lat pulldown Tailoring - Individualized as required
				Feasibility	Patient			
Kirkham	2018	Effectiveness	*n* = 73	Acceptability	Patient	Community (specialist exercise clinic) + Home	Breast cancer	What (materials) - Treadmill, elliptical, upright or recumbent cycle ergometer, resistance machines and dumbbells, Who (qualifications) - Lead by local university (lead exercise trainer, graduate exercise trainer, volunteer kinesiology student) How (delivery)- community base program was combined with home-program When, how much (dosage) - Included aerobic and resistance exercise (e.g. press, leg curls, calf raises, chest press, and seated row on machine; triceps extensions and biceps curls using dumbbells; two core-strengthening exercises). Program commenced with supervised 3x/wk. (length of chemotherapy, plus radiation if received) then reduce to 2x/wk. for 10 wks and then 1x/wk. for 10 wks during maintenance phase. Tailoring - Individualized as required; Aerobic component commenced at 20 min and increased to 30 min duration after wk. 4 (Progressive from 50 to 70% of APMHR HRR over wks 1–8, 70–75% for wks 9). Resistance commenced at 1 × 10 and then increased to 2 × 10–12 for remaining program (Chest and leg press: 50% estimated 1-RM, Similar RPE for all other exercises Weights were progressed every 4 wks up to 75% of 1-RM).
				Appropriateness	Intervention			
				Cost	Organisation + Patient			
				Feasibility	Patient			
				Penetration	Organisation			
Kirkham	2019	Effectiveness	n = 73	Appropriateness	Intervention	Community (specialist exercise clinic) + Home	Breast cancer	See Kirkham 2018
Leach	2014	Implementation	Not reported	Adoption	Organisation	Community (specialist exercise clinic) + Home	Breast cancer	What (materials) - Not reported Who (qualifications) - Initial assessment by certified exercise physiologists How (delivery) - Option of home-based or community-based (group) When, how much (dosage) - 2 days aerobic (40–60% APMHR for 20–60 min), 1 day of resistance exercise (varies between 1 and 3 sets of 8–12 repetitions and 5–14 exercises depending on difficulty level) and 5–7 days of flexibility exercise/wk. Participants are provided with 3 levels of difficulty (easy, medium, hard). Home-based exercisers are given resources that includes pictures and descriptions of all exercises and complete a fitness log to track adherence. Tailoring - Self-administered tailoring
Leach	2015	Effectiveness	*n* = 80	Acceptability	Patient	Community (specialist exercise clinic) + Home	Breast cancer	See Leach 2014
				Feasibility	Patient			
Leach	2016	Effectiveness	*n* = 63 (maintenance phase)	Feasibility	Patient	Community (specialist exercise clinic) + Home	Breast cancer	See Leach 2014
Mackenzie	2013	Effectiveness	*n* = 66	Appropriateness	Intervention	Community (specialist exercise clinic)	Any cancer type	What (materials) - Not reported Who (qualifications) - qualified yoga instructors How (delivery) – group-based in community When, how much (dosage) - 7 wk. program (1x75minute session). Combine initial breathing exercises, then 6–10 modified yoga poses, finish with relaxation exercise. Tailoring - Individualized as required
				Feasibility	Patient			
Marker	2018	Effectiveness	*n* = 170	Cost	Intervention	Community (specialist exercise clinic) + Home	Any cancer type	What (materials) - Not reported Who (qualifications) - Cancer Exercise Specialist or trained and supervised program interns completing a degree in Exercise Physiology or a related field How (delivery) - Commence with 2–3 individual session and then small group exercise When, how much (dosage) - Each session is 50 min in duration and commences with 10 min warm-up. Month one includes 2–3 individual sessions per wk., month two includes 2 group sessions (max 4 participants) per wk. and month three includes 1 group session per wk. Participants also receive unlimited access to the fitness facility during off-peak weekday hours and all day on weekends. Participants are provided with 3 levels of difficulty (easy, medium, hard). Home-based exercisers are given resources that includes pictures and descriptions of all exercises and complete a fitness log to track adherence. Exercise intensity during each session is highly adaptable and continuously adjusted Tailoring - Individualized and tailored plans
				Feasibility	Patient			
Muraca	2011	Implementation	*n* = 51	Acceptability	Patient	Hospital (out-patient) + Home	Breast cancer	What (materials) - Pedometer and resistance band Who (qualifications) - Fitness Professional (plus dietitian and social workers) How (delivery)- Group + Home-based When, how much (dosage) - 5x2hour sessions delivered over 10–12 wks. Includes a combination of diet, exercise and facilitated discussions to support behaviour change. A physical activity log is provided at the start of the program, DVD CD with 30 and 50 min audio coach-guided walking sessions. Recommendations include regular exercise initially 30 min 3–5 x/wk. Add resistance exercises Tailoring - Individualized and tailored plans
Noble	2012	Effectiveness	*n* = 386	Feasibility	Patient	Community (specialist exercise clinic)	Any cancer type	What (materials) - Polar heart rate monitor, resistance program integrates a range of equipment Who (qualifications) - Certified exercise physiologists How (delivery) - Group-based When, how much (dosage) - 2x/wk. for 1 h over 12 wks. Sessions include aerobic (progressively lengthened over 24 sessions and then increase intensity), resistance (15 reps increasing to 20 reps before weights are increased) and stretching/flexibility at the end of the session. No home exercise provided due to perceived risk. Tailoring - Individualized and modified as required based upon patient presentation.
Rajotte	2012	Effectiveness	*n* = 221	Acceptability	Patient	Community (fitness centre)	Any cancer type	What (materials) – Not reported Who (qualifications) - Personal trainer (ratio 1 trainer to 7 participants. Maximum 14 participants per group) How (delivery) - Group-based When, how much (dosage) - 2x/wk. for 12 wks (90-min sessions). 10-min aerobic warm-up, resistance exercise 50 min and 30 min ‘community building’ time. Participants and their immediate family receive a 12-wk YMCA membership. They can access the YMCA facilities on days other than the designated sessions and are encouraged to exercise outside the designated sessions. Tailoring - Individualized
				Appropriateness	Intervention			
Rogers	2019	Implementation	*n* = 30	Adoption	Organisation	Community (specialist exercise clinic or fitness centre) + Home	Breast cancer	What (materials) – Not reported, however implementation toolkit is described that supports local adaptions based upon facilities Who (qualifications) - fitness instructor or physiotherapist How (delivery) – Combined group-based program with home exercises When, how much (dosage) - 12 supervised sessions (10 in month one and 2 in month two), home-based exercise beginning in week 3 to work towards 150 min/wk. of mod/vig physical activity by the end of the 3-month intervention, coupled with 3 physical activity counselling sessions (in-person or by telephone; one in month 2 and two in month 3), and 6 group discussions (three in month 1, two in month 2, and one in month 3 Tailoring - Individualized
				Appropriateness	Intervention			
				Cost	Organisation			
Santa Mina	2012	Implementation	NA	Adoption	Organisation	Community (specialist cancer clinic) + Home	Any cancer type	What (materials) - exercise bands, a stability ball, and a yoga mat Who (qualifications) - Multidisciplinary program (exercise component provided by certified exercise physiologist) How (delivery) - primarily a home-based program but participants can participate in group-based sessions if desired When, how much (dosage) - Home-based program, supported by adherence strategies (staff communicate (by telephone or e-mail) to address barriers, exercise manual that reinforces strategies for behaviour change, access to the weekly group exercise class to facilitate social support, access to educational seminar and psychologists to support behaviour change). Weekly group sessions are 90-min duration and include a 10-min warm-up, 20 min of low-impact aerobic exercise, 20 min of resistance training, and 10 min of cool-down Tailoring - Individualized
				Cost	Organisation + Intervention			
SantaMina	2017	Effectiveness	*n* = 229	Feasibility	Patient	Community (specialist cancer clinic)	Any cancer type	What (materials) - Example - arm ergometers, treadmills, stationary cycles, mini-trampolines, and elliptical machines and resistance bands, free weights, stability balls, body bars Who (qualifications) - Physiotherapists, kinesiologists, or exercise physiologists who have completed an 8-h CancerSmart rehabilitation and exercise techniques course How (delivery) - Group-based, 2 leaders per 8–10 participant When, how much (dosage) - 30 wks exercise program (2x/wk. for 10 wks and then 1x/wk. for 20 wks). Each group session is 60 min in duration and includes aerobic interval training and resistance training, stretching, and balance exercises. Participants exercise at 50–80% of estimated heart rate range for 3–5 min and then move to musculoskeletal exercise. Cycle repeats 4–6 times with exercise recorded in patient logbook. Participants are encouraged to exercise independently, aiming to achieving 150 min of mod/vig physical activity per wk. Tailoring - Individualized programs
				Fidelity	Healthcare Provider			
Santa Mina	2019	Effectiveness/Implementation	*n* = 207	Acceptability	Patient	Home + Hospital (out-patient)	Any cancer type	What (materials) - exercise mat, resistance bands, a stability ball, and a detailed exercise program manual Who (qualifications) - Physiotherapist/Occupational Therapist (comprehensive assessment), Kinesiologists (exercise programming) How (delivery)- individual with weekly group exercise class When, how much (dosage) – aerobic component included recommendation for 150 min of mod/vig intensity/week Resistance component included 2–3 sessions/wk. of 4–10 exercises Tailoring - Individualized programs
				Adoption	Organisation			
				Appropriateness	Intervention			
Sherman	2010	Effectiveness	*n* = 162	Acceptability	Patient	Community (fitness centre)	Breast cancer	What (materials) - heated swimming pool + separate room for floor-based exercise Who (qualifications) - Encore coordinator How (delivery)- Group-based When, how much (dosage) - 1 × 8 wk. (2-h session). Sessions included low-intensity mobility and stretching exercises (20 min), and progressive hydrotherapy resistance exercises (30 min) with 5-min warm-up and cool-down. Participants are given home exercise sheets that they are encouraged to complete daily. This is reviewed weekly by the Encore coordinator. Tailoring - Not reported
				Adoption	Organisation			
				Feasibility	Patient			
Speed-Andrews	2012	Effectiveness/Implementation	*n* = 23	Feasibility	Patient	Community (specialist exercise clinic or fitness centre)	Breast cancer	What (materials) - blocks, bolsters, straps, blankets Who (qualifications) - licensed senior lyengar yoga instructor and 2 assistants who are licensed instructors. How (delivery) - Group-based When, how much (dosage) - 6 (12 classes) or 12 (22 classes) wks, 90 min/session. Tailoring - postures were based on recommendations from Geeta lyengar and adapted based on individual needs.
Swenson	2014	Effectiveness	*n* = 75	Appropriateness	Intervention	Community (specialist exercise clinic)	Any cancer type	What (materials) - treadmills, elliptical machines, upright and recumbent bikes, a Life-Fitness functional cable machine, and a walking track Who (qualifications) – Physiotherapist How (delivery) Individual assessment determined whether participants participated in individual or group sessions (maximum 4 participant) When, how much (dosage) - 8-wk program with option of 6 months maintenance. Combined aerobic exercise and strength training. Individual sessions 60 min duration. Group sessions 90 min Tailoring - Session intensity and duration were adjusted for participants according to individual physiological measures
VanGerpen	2013	Effectiveness/ Implementation	*n* = 121	Appropriateness	Intervention	Community (specialist exercise clinic)	Any cancer type	What (materials) - resistance band for home use. Stationary bike, treadmill, indoor walking track, recumbent stepper, upper body ergometer. Dumbbell, machines, resistance band. Who (qualifications) - Physiotherapist or exercise physiologist How (delivery) - Group-based (max. 12 participants per group) When, how much (dosage) - 12-wk program. 30 min aerobic exercise (5-min intervals on equipment), 30 min of either strength (rotating through equipment described above), flexibly, Pilates, yoga, relaxation or water-based exercise. Patient monitored intensity Tailoring - Not reported
Wurz	2013	Implementation	NA	Adoption	Organisation	Community (specialist exercise clinic)	Any cancer type	What (materials) – Not reported Who (qualifications) - qualified yoga instructors How(delivery)- group-based in the community When, how much (dosage) - 7 wk. program (1x75minute session). Combine initial breathing exercises, then 6–10 modified yoga poses, finish with relaxation exercise. Tailoring - Individualized

*Definition for settings*

Community

Specialist exercise clinic - physical therapy clinics, specialist cancer centres or university-based publicly accessible specialist exercise centres

Fitness centre - recreation, sport or gymnasium settings

Hospital

Inpatient - exercise delivered for people admitted to hospital

Outpatient - exercise delivered for people not admitted to hospital

Home

Prescribed exercise program that is completed at home

*Key:*

*NA* not applicable; *Wk* week; *Mod* moderate; *Vig* vigorous; *ACSM* The American College of Sports Medicine; *APMHR* Age-predicted maximal heart rate; *MHR* Maximal heart rate; *HR* Heart rate; *HRR* Heart rate reserve; *RM* Repetition maximum; *RPE* Rate of perceived exertion

A collated summary of the included studies is provided in Table 4 and highlights the diversity in study design and composition of exercise interventions. Most interventions (*n* = 26, 89.7%) included a combination of aerobic, resistance and stretching exercises. Interventions were most often delivered to people with any cancer type (*n* = 16, 55.2%), using a group-based structure (*n* = 14, 48.3%), supervised by a qualified health professional (physiotherapist, exercise physiologists) (n = 14, 48.3%) and based in a community setting (*n* = 17, 58.6%). Of the 58.6% of programs that were based in the community, 27.6% (*n* = 8) were in specialist exercise clinics and 24.1% (*n* = 7) were in fitness centres and 6.9% (*n* = 2) used a combination of specialist clinics and fitness centres. Definitions for the settings are supplied in Table 3.

**Table 4:** Summary of characteristics of included studies

Descriptive Data (range)		
Sample size range	11–1635	
Intervention duration (months)	1–9	
Contact frequency (number of exercise sessions)	4–108	
Contact time (hours)^a^	8.75–108	
Follow-up (years)	NA - 2	
	**Total studies (unique programs)**	
	**n**	**%**
**Study Design**		
Quasi- experimental	16 (14)	43.2 (45.2)
Descriptive report	8 (6)	21.6 (19.4)
Observational	7 (6)	18.9 (19.4)
Randomised control trial	3 (2)	8.1 (6.5)
Mixed methods	2 (2)	5.4 (6.5)
Qualitative	1 (1)	2.7 (3.2)
**Setting**		
Community		
Fitness centre	11 (7)	29.7 (24.1)
Specialist exercise clinic	8 (8)	21.6 (27.6)
Combined specialist exercise clinic and fitness centre	3 (2)	8.1 (6.9)
*Sub-total*	*22 (17)*	*59.5 (58.6)*
Hybrid program		
Community + Home	9 (6)	24.3 (20.7)
Hospital + Home	2 (2)	5.4 (6.9)
Combined hospital + home + community	1 (1)	2.7 (3.4)
Hospital + Community	1 (1)	2.7 (3.4)
*Sub-total*	*13 (10)*	*35.1 (34.5)*
Hospital		
Not stated outpatient and/or inpatient	1 (1)	2.7 (3.4)
Outpatient	1 (1)	2.7 (3.4)
Inpatient	0 (0)	0.0 (0.0)
*Sub-total*	*2 (2)*	*5.4 (6.9)*
Home-program	0 (0)	0.0 (0.0)
**Cancer Type**		
Any cancer type	19 (16)	51.4 (55.2)
Breast Cancer	11 (8)	29.7 (27.6)
Prostate Cancer	5 (3)	13.5 (10.3)
Not specified	2 (2)	5.4 (6.9)
**Intervention Type**		
Mixed aerobic/resistance/stretching	32 (26)	86.5 (89.7)
Yoga	3 (2)	8.1 (6.9)
Football (soccer)	2 (1)	5.4 (3.4)
**Intervention Delivery**		
Group	20 (14)	54.1 (48.3)
Combination	14 (12)	37.8 (41.4)
Not reported	2 (2)	5.4 (6.9)
Individual	1 (1)	2.7 (3.4)
**Staff delivering the Intervention**		
Qualified health professional (physiotherapy)	17 (14)	45.9 (48.3)
Fitness professional	10 (9)	27.0 (31.0)
Varied (qualified health professional + fitness professionals)	6 (4)	16.2 (13.8)
Not reported	4 (2)	10.8 (6.9)
Nurse/Medical professional	0 (0)	0.0 (0.0)

^a^ based on studies that included time

The results for each implementation outcome and study type are summarised in Table 5. The most common implementation outcomes assessed were *feasibility* (*n* = 17, 54.8%) and *adoption* (*n* = 14, 45.2%) of exercise interventions. The most common classification was effectiveness study (*n* = 15, 48.4%).

**Table 5:** Synthesis of implementation outcomes and study classification across included studies

***Study***	***Implementation Outcomes***								***Study classification***		
*First author & Year*	*Acceptability*	*Adoption*	*Appropriateness*	*Cost*	*Feasibility*	*Fidelity*	*Penetration*	*Sustainability*	*Effectiveness*	*Implementation*	*Both*
Beidas 2014 [56]		X	X	X							X
Bjerre 2018 [45]			X	X	X	X			X		
Bjerre 2019 [57]			X						X		
Bultijnck 2018 [58]		X								X	
Brown 2019 [46]					X	X			X		
Cheifetz 2014 [47]					X	X					X
Cheifetz 2015 [59]					X						X
Culos-Reed 2018 [48]			X	X	X	X					X
Culos-Reed 2019 [60]				X						X	
Dalzell 2017 [61]		X		X						X	
Dennett 2017		X								X	
Dolan 2018 [62]			X		X				X		
Haas 2011 [63]		X		X	X					X	
Haas 2012 [64]					X			X	X		
Heston 2015 [65]		X		X	X	X				X	
Irwin 2017 [49]		X			X				X		
Kimmel 2014		X			X					X	
Kirkham 2016 [66]				X	X				X		
Kirkham 2018 [67]	X		X	X	X		X		X		
Kirkham 2019 [68]			X						X		
Leach 2014 [69]		X								X	
Leach 2015 [70]	X				X				X		
Leach 2016 [71]					X				X		
Mackenzie 2013 [51]			X		X				X		
Marker 2018 [72]				X	X				X		
Muraca 2011 [73]	X									X	
Noble 2012 [52]					X				X		
Rajotte 2012 [53]	X		X						X		
Rogers 2019 [74]		X	X	X						X	
Santa Mina 2012 [75]		X		X						X	
Santa Mina 2017 [54]					X	X			X		
Santa Mina 2019 [76]	X	X	X								X
Sherman 2010 [77]	X	X			X				X		
Speed-Andrews 2012 [78]					X						X
Swenson 2014 [79]			X						X		
VanGerpen 2013 [80]			X								X
Wurz 2013 [55]		X								X	
**TOTAL (*n*** **= 37)**	**6 (16.2%)**	**14 (37.8%)**	**13 (35.1%)**	**12 (32.4%)**	**21 (56.8%)**	**6 (16.2%)**	**1 (2.7%)**	**1 (2.7%)**	**18 (48.6%)**	**12 (32.4%)**	**7 (18.9%)**
**TOTAL (unique programs) (n = 31)**	**6 (19.4%)**	**14 (45.2%)**	**11 (35.5%)**	**11 (35.5%)**	**17 (54.8%)**	**6 (19.4%)**	**1 (3.2%)**	**1 (3.2%)**	**15 (48.4%)**	**11 (35.5%)**	**6 (19.4%)**

The results are expanded upon in Supplementary Table 5 and below.

### Acceptability

Six studies reported on the acceptability of exercise interventions for people with cancer, measured at the patient-level [53, 67, 70, 73, 76, 77]. Patient satisfaction (variously defined as enjoying the program, finding the program useful/valuable) was generally high, with five studies reporting acceptability levels above 80% [53, 70, 73, 76, 77]. None of the included studies reported on the acceptability of exercise interventions measured at the healthcare professional level.

### Adoption

Fourteen studies reported on exercise intervention adoption [49, 50, 55, 56, 58, 61, 63, 65, 69, 74–77, 81]. Nine studies assessed qualitative barriers and enablers to intervention adoption (refer to supplementary Table 5) but did not measure adoption [50, 55, 56, 61, 69, 74–76, 81]. Four studies explored uptake by organisations [49, 63, 65, 77] and one study assessed both organisational uptake and qualitative barriers to adoption [58]. Of the five studies that measured adoption, two reported the percentage of organisations across the country who had adopted exercise oncology programs, with 60% of hospitals in Belgium adopting programs and 18% of YMCA’s in America delivering a specific program (i.e., Livestrong at the YMCA). The three further studies that measured organisation adoption rates provided the raw number of organisations delivering a program, without reference to total possible delivery organisations (i.e., 40 sites across Australia). None of the identified studies reported on overall program uptake rates by healthcare providers, such as the total number of professionals making patient referrals to exercise.

### Appropriateness

Thirteen studies reported on the appropriateness of exercise interventions [45, 48, 51, 53, 56, 57, 62, 67, 68, 74, 76, 79, 80], representing 11 unique programs. Six studies [45, 51, 56, 57, 62, 74] reported that appropriateness was established by testing the efficacy of the exercise intervention in the target population (in a previous efficacy trial). Five studies [48, 67, 68, 79, 80] reported using multiple data sources (including a literature review, reference to established models of care and/or review of barriers and enablers) to establish appropriateness, with only two of these studies directly engaging with program staff through the development phase [48, 80]. Two studies stated a phased approach to implementation (a pilot period completed prior to full intervention roll-out) was undertaken to establish appropriateness of the intervention [53, 76].

### Cost

Twelve studies reported on costs associated with implementation [45, 48, 56, 60, 61, 63, 65–67, 72, 74, 75], representing 11 unique programs. Two studies estimated the intervention implementation costs in the set-up year (e.g., purchase of computers and equipment, cleaning, personnel), stating that it cost $US44,821 and $US46,213, respectively [45, 67]. One study reported the implementation cost to be approximately $350 per participant [74]. Four studies reported that philanthropic donations were used to support the ongoing organisational costs associated with the exercise intervention [61, 63, 65, 75]. Hybrid models of funding subsided the costs associated with intervention use, including a mix of fee-for-service (upfront, set cost per session) and subsidised costs (total session costs off-set through donations, sponsorship) [48, 56, 60, 63, 65, 66, 72, 75]. Studies from the United States and Canada were the only ones to report on costs, where costs were measured as direct healthcare costs.

### Feasibility

Twenty-one studies reported on the feasibility of delivering interventions, operationalised as either attendance and/or attrition rates for the exercise interventions [45–52, 54, 59, 62–67, 70–72, 77, 78], representing 17 unique programs. The attrition rates ranged from 22 to 56% across nine studies, with measurement of program discontinuation occurring between time ranges of 12 weeks to 6 months. The mean attrition rate for exercise intervention was 38.4% (*n* = 7) [46, 47, 50, 52, 59, 63, 64, 67, 77]. The attendance rates ranged from 30 to 83% across 16 studies. The mean attendance rate was calculated as 63.7% (*n* = 15) [45, 46, 48, 49, 51, 54, 62, 65–67, 70–72, 77, 78].

### Fidelity

Six studies reported aspects of fidelity were monitored with reference to a documented pre-planned protocol for exercise and cancer [45–48, 54, 65]. Fidelity is typically measured by comparing the original protocol to what is delivered according to: 1) adherence to the protocol, 2) dose or amount of program (e.g., frequency, duration) delivered (with consideration of the core components that establish intervention effectiveness) and 3) quality of program delivery [82]. One study measured both adherence and quality of the program and stated adherence by football coaches to deliver the intervention as per the documented protocol was approximately 76%, and program quality was achieved through training staff [45]. A further five studies reported that the quality of program delivery was achieved through staff training and/or achieving certification to deliver their program as prescribed [46–48, 54, 65]. No studies were identified that monitored the amount of program delivery with respect to the pre-planned protocol.

### Penetration

One study reported on exercise intervention penetration, which was defined as patients referred to the intervention reported with consideration to total eligible patient population [67]. This study, which evaluated the implementation of an exercise intervention for people diagnosed with breast cancer, reported that 53% of eligible patients were referred to the program [67].

### Sustainability

One study reported on the sustainability of the exercise intervention within the organisational setting [64]. The authors also collected secondary outcome data about sustainability at the patient level, defined as whether the exercise sustained (> 12 months) the desired health outcomes for the patient [64]. Sustaining the program as part of normal organisational operations was attributed to addressing common challenges people diagnosed with cancer face in being active. This included providing tailored exercise by trained staff and establishing a not-for-profit entity to provide these services for free in the community [64]. The secondary outcome identified that the exercise intervention was effective in sustaining improvements to quality of life for patients [64].

### Quality assessment

The quality of included studies explored through this review varied (refer to Supplementary Table 2). Studies were generally downgraded because they were not sufficiently powered to allow confidence in the inferences drawn about whole populations, and/or they failed to document possible differences between groups based on participants lost to follow-up. Further, many (64.5%, *n* = 6) of the studies classified as implementation studies were descriptive, with no objective measure of the implementation outcomes.

## Discussion

This review identifies that exercise interventions are being implemented for people diagnosed with cancer using pragmatic study designs, but there is no consensus about how successful implementation should be defined, measured, and reported. Measuring implementation outcomes, using an established framework, can generate new knowledge in this area by conceptualising and defining what constitutes success [33]. To the best of our knowledge, this is the first systematic review that has explored implementation outcomes in exercise and cancer using the Implementation Outcomes Framework [30]. The included studies represent diverse interventions that are delivered across different settings and for various cancer types. For example, interventions involving yoga, sport, aerobic and resistance exercises were identified. These interventions were delivered in communities or hospitals and program eligibility (based on cancer diagnosis) varied across patient sub-type to include any cancer type through to being limited to a specific cancer type. Most studies adopted a quasi-experimental design applied to test effectiveness of the intervention, with descriptive designs more common in studies classified as implementation. The implementation outcomes that were most frequently assessed in the eligible studies were feasibility and adoption. Furthermore, the fidelity to intervention delivery is infrequently reported and the true cost of implementation is relatively unknown. Penetration and sustainability were the least frequently assessed implementation outcomes.

Almost 60% of included studies measured feasibility. Feasibility may have been measured more often than other implementation outcomes because of the interdependence with the clinical outcomes of exercise interventions (e.g., patients must adhere to the intervention to derive the desired clinical effect) and the ease of collection (e.g., staff can record attendance levels). It was also one of the few implementation outcomes that was explored at the patient-level by reporting patient attendance and/or attrition rates, recognising that factors at levels other than the patient can influence this outcome (e.g., resources provided by the organisation or expertise of the healthcare providers). Almost half the studies in this review were classified as effectiveness studies. Effectiveness studies typically focus on patient outcomes [83], conferring a focus on patient-level outcomes in included studies. Whilst outside the scope of this review, future studies should explore the feasibility of exercise interventions for other stakeholders such as those who assume non-clinical roles [84]. For example, this might apply to health administrators who fund exercise interventions and policy makers who establish the strategic policy environment in cancer care. Feasibility of exercise programs for program co-ordinators has been explored in the Canadian setting [84], however more research is needed. Successful implementation involves multiple stakeholders and whilst exercise services appear feasible for patients, it may not be feasible for funders or policy makers. This would also improve consistency with Proctor’s definition of feasibility which suggests measurement at provider, organisation or setting level [30].

Some aspects of adoption were evaluated in the included studies, including the barriers and enablers that impact implementation and organisational uptake rates. Despite this, no studies were identified that measured overall adoption rates by healthcare providers. Measuring the proportion of healthcare providers that adopt the intervention could provide better insights into referral patterns through identifying who is making (and not making) referrals. Further, only the study by Rogers and colleagues [74] applied an implementation science framework to collate the adoption barriers and enablers. Implementation science frameworks can guide the comprehensive compilation of factors that influence implementation [32]. Subsequent research should build on the work of Rogers and colleagues to identify and test the effectiveness (and cost) of different strategies that can mitigate common implementation barriers. This may include the effectiveness of different implementation strategies that can facilitate systematic, routine referral by healthcare providers. A recently validated questionnaire completed by healthcare providers may assist in identifying relevant strategies specific to cancer and exercise [85].

Including a cost evaluation for these strategies would address another gap identified through this review. No studies were identified that measured the cost of implementation strategies. Providing this information would enable policymakers to make astute decisions about the sustainable funding of exercise interventions. Further, evidence suggests implementation strategies, such as staff training, can increase the likelihood of successful implementation [86, 87]. Implementation strategies are the actions undertaken designed to cause the change that produces the desired implementation outcome [88]. Conceptually, within implementation research they are the elements that sit between the intervention and the outcome and are the focus of empirical testing [89]. Most of the articles categorised as implementation in this review were descriptive and did not empirically test implementation strategies. Further, of the 37 included articles, only three were randomised control trials (representing 2 unique programs) and were described as effectiveness trials. Whilst the utility of randomised control trials for implementation research is contested [90], there is a need for implementation studies that use experimental designs to rigorously test strategies [91].

Another important finding established through this review was that fidelity is infrequently measured, with the quality of program delivery most frequently applied. Whilst accurately measuring fidelity is a challenge [82], it typically considers compliance with the intervention protocol and adaptions to this protocol (based on the setting, population). Compliance with the intervention protocol was difficult to establish. Most studies (*n* = 25, 80.6%) in this review were tailored which is recommended (at the individual level) to ensure exercise programs are suitable for participants [13]. What remains unclear is the extent and type of tailoring of intervention components and whether this extended to significant changes to the intervention which could be considered as ‘adaptions’ to the core elements of the program (consistent with Proctor’s definition). Without this information it is difficult to accurately measure the fidelity of program delivery. More detailed reporting in future studies about how tailoring alters an intervention is needed and whether these changes extended to significant program adaptions and any impact on fidelity of delivery should be specified. For example, the review by Beidas and colleagues reported three changes to their program (training staff, adding a program co-ordinator and implementing a phone call reminder to increase uptake of the program) [56] which was part of a barrier and enabler analysis but is not related back to measuring an implementation outcome such as fidelity.

A major finding of this review relates to the later stages of implementation. Very few studies evaluated penetration and sustainability, indicating limited knowledge about how exercise interventions are continued after initial implementation efforts cease. Evidence suggests that many interventions are not sustained, or only parts of an intervention are sustained [40]. This can contribute to resource waste and delivery of ineffective interventions. More research is needed to investigate how interventions are integrated within organisational activities and sustained over time. This is particularly important given that sustaining interventions is a dynamic process that requires repeated and continued attention [92].

This review was guided by the Implementation Outcomes Framework. Other studies in exercise and cancer have used similar outcomes frameworks to explore the translation potential of exercise interventions based in the community [36], for specific cancer type (breast cancer) [93, 94] and to explore sustainability of interventions [95]. Like Jankowski and colleagues [95], our review confirmed a paucity of research that explores organisational-level factors that impact on sustainability of exercise interventions. However, our review does extend current knowledge beyond identifying adoption barriers and enablers [93] and organisation uptake rates [36] by exploring overall adoption rates of healthcare providers. Additionally, previous research has produced contrary results regarding reach and study participants representativeness of the broader population [93, 94]. The one study that measured penetration in this review found differences (in intervention reach) between those who were referred and those who were not referred to the intervention [67], suggesting a possible referral bias. Furthermore, despite the gaps in measuring and reporting implementation outcomes, effectiveness/implementation study protocols were identified through the screening process that plan to incorporate these outcomes [96–98]. This suggests researchers are recognising the value of measuring successful implementation using established outcome frameworks [30, 99]. This type of research will support the translation of research findings into practice, as proposed by the ACSM and other international health organisations.

This review is not without limitations. It was challenging to capture all relevant studies because of the inconsistencies in terminology. For example, in cancer care settings exercise may be included within a rehabilitation program, however we did not include rehabilitation as a search-term due to its generic nature. Several strategies were employed to overcome inconsistencies in terminology, including hand-searching the *Moving through Cancer* exercise program registry. A second limitation of this review was associated with delineating between efficacy and effectiveness studies. An existing categorisation was used to define studies [100], however some studies that were described by the authors as pragmatic employed methods synonymous with efficacy studies and were therefore excluded. Further, there is a lack of quality assessment tools that are designed specifically for implementation study designs. This resulted in some of the standard quality assessment items being not applicable to the eligible studies. Third, we excluded studies where people were specifically receiving end-of-life care, as distinct from long-term maintenance therapies. Finally, this review identified relatively few unique exercise interventions that were exclusive categorised as either effectiveness or implementation studies. In some cases, single programs were evaluated at multiple time points leading to multiple publications on the same program. As such, caution should be used when drawing conclusions from these findings.

The review results suggest exercise interventions may be successfully implemented, however relatively little information is published about how successful implementation is defined, measured and reported. This review examined all of Proctor et al. implementation outcomes. Future work should build on this review by investigating each implementation outcome in greater detail and across all levels of implementation (such as healthcare provider, organisation, and policy level). Currently, little data exist to: 1) quantify how many providers are adopting exercise interventions; 2) identify what portion of total eligible patient population are being referred to interventions; 3) define the total cost of implementation (including the cost of implementation strategies); and 4) understand how to sustain interventions over time. These outcomes become more valuable as we shift attention to those implementation strategies used in practice. Augmenting measures with qualitative data about how these outcomes were achieved is also required. This is particularly evident with feasibility, where outcomes varied despite high level of measurement. Further understanding how some interventions achieved higher levels of attendance/reduced attrition is required. The actions that lead to these outcomes should then be considered for replication in future implementation efforts. To conclude, measuring and evaluating implementation outcomes in cancer and exercise offers enormous potential to help conceptualise what is ‘implementation success’. It paves the way to develop (and subsequently test) causal relationships between the exercise interventions, the strategies or tools used during implementation and the outcome achieved [101]. Only then will researchers in exercise and cancer begin to unpack the implementation process and explain ‘how and why’ implementation was successful.

## Supplementary Information

**Additional file 1: Supplementary Table 1.** Search Strategy.

**Additional file 2: Supplementary Table 2.** Definitions of terms for study classification [102–104].

**Additional file 3: Supplementary Table 3.** Quality Assessment.

**Additional file 4: Supplementary Table 4.** Excluded Studies.

**Additional file 5: Supplementary Table 5.** Summary of results Implementation Outcomes.

## Data Availability

All data generated or analysed during this study are included in this published article and its supplementary information files.

## Abbreviations

ACSM: American College of Sports Medicine
CERT: Consensus on Exercise Reporting Template
CPGs: Clinical practice guidelines
JBI: Joanna Briggs Institute
PRISMA: Preferred Reporting Items for Systematic reviews and Meta-Analysis
