# Supplementary material for: Exercise interventions for people diagnosed with cancer: a systematic review of implementation outcomes
Source: BMC Cancer. 2021 May 30;21:643. doi: 10.1186/s12885-021-08196-7 (PMC8166065; doi:10.1186/s12885-021-08196-7)
Supplement: Supplementary file 1 — Additional file 1: Supplementary Table 1. Search Strategy. [file 12885_2021_8196_MOESM1_ESM.docx]

**Supplementary Table 1: Search Strategy**

|  | **Cancer** | **Exercise** | **Implementation outcomes** | **Evidence** |
| --- | --- | --- | --- | --- |
| **Medline Title/Abstract Terms** | cancer OR malignan* OR oncolog* OR neoplasm* OR tumor OR tumour OR carcinoma OR chemotherapy OR radiotherapy OR androgen deprivation OR mastectomy OR lumpectomy OR pneumonectomy OR lobectomy OR colectomy OR bowel resection OR prostatectomy OR laryngectomy OR esophagectomy OR gastrectomy | exercis* OR "weight lifting" OR sport* OR "strength training" OR "resistance training" OR aerobic OR walk* OR fitness OR “physical activit*” | (Acceptab* Or Satisf*) OR (Adopt* OR Uptake OR utili*OR implement* OR “intention to try” OR barrier* OR enable* OR facilitate*)  OR (Appropriat* OR “perceived fit” OR relevan* OR compat* OR suitab* OR useful* OR practica*) OR (cost* OR economic* OR finance*) OR (Feasibil*OR Utili* OR Practica*) OR (Fidelity OR Integrity OR “delivered as intended” OR adhere* OR “quality of program delivery”) OR (Penetrat* OR integrat* OR “spread” OR “service access”) OR (Sustain* OR maintenance OR continu* OR durab* OR incorporate*OR integrat* OR institutionaliz* OR maintain* OR routin*OR institutionalis*) | Innovat* OR EBP OR evidence* OR empirical* OR evaluat* |
| **Medline**  **MeSH Terms** | (MH "Neoplasms") OR (MH "Radiation Oncology") OR (MH "Medical Oncology") | (MH "Exercise Therapy") OR (MH "Exercise") OR (MH "Plyometric Exercise") OR (MH "Resistance Training") OR (MH "Physical Conditioning, Human") OR (MH "Muscle Stretching Exercises") OR (MH "Circuit-Based Exercise") OR (MH "Exercise Movement Techniques") OR (MH "Walking") OR (MH "Weight Lifting") OR (MH "Sports") | (MH "Patient Satisfaction") OR (MH "Intention") OR (MH "Costs and Cost Analysis") OR (MH "Cost-Benefit Analysis") OR (MH "Cost Savings") OR (MH "Cost of Illness") OR (MH "Quality Control") OR (MH “Delivery of health care”) OR (MH ”Comprehensive health care”) OR (“MH “Quality of health care”) | (MH "Evidence-Based Practice") OR (MH "Evidence-Based Medicine") OR (MH “diffusion of innovation”) OR (MH "Program Evaluation") |
