# Supplementary material for: Exercise interventions for people diagnosed with cancer: a systematic review of implementation outcomes
Source: BMC Cancer. 2021 May 30;21:643. doi: 10.1186/s12885-021-08196-7 (PMC8166065; doi:10.1186/s12885-021-08196-7)
Supplement: Supplementary file 2 — Additional file 2: Supplementary Table 2. Definitions of terms for study classification [102–104]. [file 12885_2021_8196_MOESM2_ESM.docx]

**Supplementary Table 2: Definitions of terms for study classification**

| **Term** | **Definition** | **Categorisation in this paper** |
| --- | --- | --- |
| **Pilot study** | Pilot studies are a version of the main study that is run in miniature to test whether the components of the main study can work together. It is focused on ensuring that the processes of the main study (e.g., recruitment, randomisation, treatment, and follow-up assessments) all run smoothly [102] | **Excluded** |
| **Efficacy study** | Efficacy trials (explanatory trials) determine whether an intervention produces the expected result under ideal circumstances. [103] | **Excluded** |
| **Effectiveness** | Determines the impact of an intervention with demonstrated efficacy when it is delivered under ‘real-world’ conditions [81] | **Effectiveness** |
| **Implementation** | The process of putting to use or integrating evidence-based interventions within a setting [81] | **Implementation** |
| **Implementation research** | Scientific study of the use of strategies to adopt and integrate evidence-based health interventions into clinical and community settings to improve individual outcomes and benefit population health. [104] | **Implementation** |
