# Supplementary material for: Exercise interventions for people diagnosed with cancer: a systematic review of implementation outcomes
Source: BMC Cancer. 2021 May 30;21:643. doi: 10.1186/s12885-021-08196-7 (PMC8166065; doi:10.1186/s12885-021-08196-7)
Supplement: Supplementary file 3 — Additional file 3: Supplementary Table 3. Quality Assessment. [file 12885_2021_8196_MOESM3_ESM.docx]

**Supplementary Table 3: Quality Assessment**

*Mixed methods*

| **Author** | **Year** | **Are there clear research questions?** | **Do the collected data allow to address the research questions?** | **Is there an adequate rationale for using a mixed method design to address the research question?** | **Are the different components of the study effectively integrated to answer the research question?** | **Are the outputs of the integration of qualitative and quantitative components adequately interpreted?** | **Are divergences and inconsistencies between quantitative and qualitative results adequately addressed?** | **Do the different components of the study adhere to the quality criteria of each tradition of the methods involved?** |
| --- | --- | --- | --- | --- | --- | --- | --- | --- |
| Beidas | 2014 | Yes | Yes | Yes | Yes | Yes | Unclear | Yes |
| Dennett | 2017 | Yes | Yes | Yes | Unclear | Unclear | No | Yes |

*Cross-sectional*

| **Author** | **Year** | **Were the criteria for inclusion in the sample clearly defined?** | **Were the study subjects and the setting described in detail?** | **Was the exposure measured in a valid and reliable way?** | **Were objective, standard criteria used for measurement of the condition?** | **Were confounding factors identified?** | **Were strategies to deal with confounding factors stated?** | **Were the outcomes measured in a valid and reliable way?** | **Was appropriate statistical analysis used?** |
| --- | --- | --- | --- | --- | --- | --- | --- | --- | --- |
| Bultijnck | 2018 | Unclear | Yes | Yes | Yes | Not applicable | Not applicable | Yes | Yes |

*Quasi-experimental*

| **Author** | **Year** | **Is it clear in the study what is the ‘cause’ and what is the ‘effect’ (i.e. there is no confusion about which variable comes first)?** | **Were the participants included in any comparisons similar?** | **Were the participants included in any comparisons receiving similar treatment/care, other than the exposure or intervention of interest?** | **Was there a control group?** | **Were there multiple measurements of the outcome both pre and post the intervention/exposure** | **Was follow up complete and if not, were differences between groups in terms of their follow up adequately described and analyzed?** | **Were the outcomes of participants included in any comparisons measured in the same way?** | **Were outcomes measured in a reliable way?** | **Was appropriate statistical analysis used?** |
| --- | --- | --- | --- | --- | --- | --- | --- | --- | --- | --- |
| Brown | 2019 | Yes | Yes | Yes | No | Yes | Unclear | Yes | Yes | Yes |
| Culos-Reed | 2019 | Yes | Yes | Yes | No | Yes | Yes | Yes | Yes | Unclear |
| Dolan | 2018 | Yes | Yes | Yes | No | Yes | Yes | Yes | Yes | Yes |
| Haas | 2012 | Yes | Yes | Yes | No | Yes | Yes | Yes | Yes | Unclear |
| Kirkham | 2018 | Yes | Yes | Yes | No | Yes | Unclear | Yes | Yes | Yes |
| Kirkham | 2019 | Yes | Yes | Yes | No | Yes | Unclear | Yes | Yes | Yes |
| Leach | 2015 | Yes | Yes | Yes | No | Yes | Unclear | Yes | Yes | Unclear |
| Leach | 2016 | Yes | Yes | Unclear | No | Yes | Yes | Yes | Yes | Yes |
| MacKenzie | 2013 | Yes | Unclear | Yes | No | Yes | Unclear | Yes | Yes | Yes |
| Muraca | 2011 | Not applicable | Yes | Unclear | No | No | No | Not applicable | Unclear | Unclear |
| Rajotte | 2012 | Yes | Yes | Yes | No | Yes | Yes | Yes | Yes | Yes |
| Santa Mina | 2019 | Yes | Yes | Yes | No | Yes | No | Yes | Yes | Yes |
| Sherman | 2010 | Yes | Unclear | Yes | Yes | Yes | Yes | Yes | Unclear | Unclear |
| Speed-Andrews | 2012 | Yes | Yes | Yes | No | Unclear | Not applicable | Yes | Yes | No |
| Swenson | 2014 | Yes | Yes | Unclear | No | Yes | Yes | Yes | Yes | Unclear |
| VanGerpen | 2013 | Yes | Yes | Yes | No | Yes | No | Yes | No | Unclear |

*Text and opinion*

| **Author** | **Year** | **Is the source of the opinion clearly identified?** | **Does the source of opinion have standing in the field of expertise?** | **Are the interests of the relevant population the central focus of the opinion?** | **Is the stated position the result of an analytical process, and is there logic in the opinion expressed?** | **Is there reference to the extant literature?** | **Is any incongruence with the literature/sources logically defended?** |
| --- | --- | --- | --- | --- | --- | --- | --- |
| Dazell | 2015 | Yes | Yes | Yes | Yes | Yes | Not applicable |
| Haas | 2011 | Yes | Yes | Yes | Yes | Yes | Not applicable |
| Heston | 2018 | Yes | Yes | Yes | Yes | Yes | Not applicable |
| Kimmel | 2014 | Yes | Yes | Yes | Yes | Yes | Not applicable |
| Leach | 2014 | Yes | Yes | Yes | Yes | Yes | Not applicable |
| Rogers | 2019 | Yes | Yes | Yes | Yes | Yes | Not applicable |
| Santa Mina | 2012 | Yes | Yes | Yes | Yes | Yes | Not applicable |
| Wurz | 2013 | Yes | Yes | Yes | Yes | Yes | Not applicable |

*Case series*

| **Author** | **Year** | **Were there clear criteria for inclusion in the case series?** | **Was the condition measured in a standard, reliable way for all participants included in the case series?** | **Were valid methods used for identification of the condition for all participants included in the case series?** | **Did the case series have consecutive inclusion of participants?** | **Did the case series have complete inclusion of participants?** | **Was there clear reporting of the demographics of the participants in the study?** | **Was there clear reporting of clinical information of the participants?** | **Were the outcomes or follow up results of cases clearly reported?** | **Was there clear reporting of the presenting site(s)/clinic(s) demographic information?** | **Was statistical analysis appropriate?** |
| --- | --- | --- | --- | --- | --- | --- | --- | --- | --- | --- | --- |
| Kirkham | 2016 | Yes | Yes | Yes | Yes | Yes | Yes | Yes | Yes | Yes | Yes |
| Marker | 2018 | Yes | Yes | Yes | No | Yes | Yes | Yes | Yes | Yes | Yes |
| Noble | 2012 | Yes | Yes | Unclear | Yes | No | Yes | Yes | Yes | Yes | Unclear |

*Cohort*

| **Author** | **Year** | **Were the two groups similar and recruited from the same population?** | **Were the exposures measured similarly to assign people to both exposed and unexposed groups?** | **Was the exposure measured in a valid and reliable way?** | **Were confounding factors identified?** | **Were strategies to deal with confounding factors stated?** | **Were the groups/participants free of the outcome at the start of the study (or at the moment of exposure)?** | **Were the outcomes measured in a valid and reliable way?** | **Was the follow up time reported and sufficient to be long enough for outcomes to occur?** | **Was follow up complete, and if not, were the reasons to loss to follow up described and explored?** | **Were strategies to address incomplete follow up utilized?** | **Was appropriate statistical analysis used?** |
| --- | --- | --- | --- | --- | --- | --- | --- | --- | --- | --- | --- | --- |
| Cheifetz | 2014 | No | No | Yes | Yes | Yes | Unclear | Yes | Yes | Yes | Yes | Yes |
| Cheifetz | 2015 | No | No | Yes | Yes | Yes | Unclear | Yes | Yes | Yes | Yes | Yes |
| Santa Mina | 2017 | Not applicable | Yes | Unclear | Not applicable | Not applicable | Yes | Yes | Yes | Yes | Yes | Yes |

*Qualitative*

| **Author** | **Year** | **Is there congruity between the stated philosophical perspective and the research methodology?** | **Is there congruity between the research methodology and the research question or objectives?** | **Is there congruity between the research methodology and the methods used to collect data?** | **Is there congruity between the research methodology and the representation and analysis of data?** | **Is there congruity between the research methodology and the interpretation of results?** | **Is there a statement locating the researcher culturally or theoretically?** | **Is the influence of the researcher on the research, and vice- versa, addressed?** | **Are participants, and their voices, adequately represented?** | **Is the research ethical according to current criteria or, for recent studies, and is there evidence of ethical approval by an appropriate body?** | **Do the conclusions drawn in the research report flow from the analysis, or interpretation, of the data?** |
| --- | --- | --- | --- | --- | --- | --- | --- | --- | --- | --- | --- |
| Culos-Reed | 2019 | Yes | Yes | Yes | Yes | Yes | No | No | Yes | Yes | Yes |

*Randomised control trial*

| **Author** | **Year** | **Was true randomization used for assignment of participants to treatment groups?** | **Was allocation to treatment groups concealed?** | **Were treatment groups similar at the baseline?** | **Were participants blind to treatment assignment?** | **Were those delivering treatment blind to treatment assignment?** | **Were outcomes assessors blind to treatment assignment?** | **Were treatment groups treated identically other than the intervention of interest?** | **Was follow up complete and if not, were differences between groups in terms of their follow up adequately described and analyzed?** | **Were participants analyzed in the groups to which they were randomized?** | **Were outcomes measured in the same way for treatment groups?** | **Were outcomes measured in a reliable way?** | **Was appropriate statistical analysis used?** |
| --- | --- | --- | --- | --- | --- | --- | --- | --- | --- | --- | --- | --- | --- |
| Irwin | 2017 | Yes | No | Yes | Unclear | No | Unclear | Yes | Yes | Yes | Yes | Yes | Yes |
| Bjerre | 2018 | Yes | Yes | Unclear | No | No | Yes | Yes | Yes | Yes | Yes | Yes | Yes |
| Bjerre | 2019 | Yes | Yes | Unclear | No | No | Yes | Yes | Yes | Yes | Yes | Yes | Yes |
