# Supplementary material for: Exercise interventions for people diagnosed with cancer: a systematic review of implementation outcomes
Source: BMC Cancer. 2021 May 30;21:643. doi: 10.1186/s12885-021-08196-7 (PMC8166065; doi:10.1186/s12885-021-08196-7)
Supplement: Supplementary file 4 — Additional file 4: Supplementary Table 4. Excluded Studies. [file 12885_2021_8196_MOESM4_ESM.docx]

**Supplementary Table 4: Excluded Studies**

| **Author** | **Year** | **Title** | **Reason for exclusion** |
| --- | --- | --- | --- |
| Abbott, Linda; Hooke, Mary Catherine | 2017 | Energy Through Motion: An Activity Intervention for Cancer-Related Fatigue in an Ambulatory Infusion Center | 1 |
| Adams, B. E.; Yochem, A.; Minick, K.; Brennan, G. P. | 2018 | A Comparison of outcomes for a standardized versus nonstandardized physical therapy protocol in an acute care bone marrow transplant unit | 1 |
| Adamsen, Lis; Midtgaard, Julie; Rorth, Mikael; Borregaard, Niels; Andersen, Christina; Quist, Morten; Møller, Tom; Zacho, Morten; Madsen, Jan K.; Knutsen, Lasse | 2003 | Feasibility, physical capacity, and health benefits of a multidimensional exercise program for cancer patients undergoing chemotherapy | 2 |
| Adamsen, Lis; Quist, Morten; Midtgaard, Julie; Andersen, Christina; Møller, Tom; Knutsen, Lasse; Tveterås, Anders; Rorth, Mikael | 2006 | The effect of a multidimensional exercise intervention on physical capacity, well-being and quality of life in cancer patients undergoing chemotherapy | 2 |
| Alibhai, Shabbir Mh; Santa Mina, Daniel; Ritvo, Paul; Sabiston, Catherine; Krahn, Murray; Tomlinson, George; Matthew, Andrew; Segal, Roanne; Warde, Padraig; Durbano, Sara; O'Neill, Meagan; Culos-Reed, Nicole; Alibhai, Shabbir M. H. | 2015 | A phase II RCT and economic analysis of three exercise delivery methods in men with prostate cancer on androgen deprivation therapy | 2 |
| Alibhai, Shabbir M. H.; Santa Mina, Daniel; Ritvo, Paul; Tomlinson, George; Sabiston, Catherine; Krahn, Murray; Durbano, Sara; Matthew, Andrew; Warde, Padraig; O'Neill, Meagan; Timilshina, Narhari; Segal, Roanne; Culos-Reed, Nicole | 2019 | A phase II randomized controlled trial of three exercise delivery methods in men with prostate cancer on androgen deprivation therapy | 2 |
| Amritanshu, Ram R.; Rao, Raghavendra Mohan; Nagaratna, Raghuram; Veldore, Vidya Harini; Rani, Usha; Gopinath, Kodaganur S.; Ajaikumar, B. S. | 2017 | Effect of Long-term Yoga Practice on Psychological outcomes in Breast Cancer Survivors | 2 |
| Ammitzbøll, Gunn; Lanng, Charlotte; Kroman, Niels; Zerahn, Bo; Hyldegaard, Ole; Kaae Andersen, Klaus; Johansen, Christoffer; Dalton, Susanne Oksbjerg | 2017 | Progressive strength training to prevent LYmphoedema in the first year after breast CAncer – the LYCA feasibility study | 2 |
| Andersen, C.; Adamsen, L.; Moeller, T.; Midtgaard, J.; Quist, M.; Tveteraas, A.; Rorth, M. | 2006 | The effect of a multidimensional exercise programme on symptoms and side-effects in cancer patients undergoing chemotherapy--the use of semi-structured diaries | 2 |
| Arem, Hannah; Sorkin, Mia; Cartmel, Brenda; Fiellin, Martha; Capozza, Scott; Harrigan, Maura; Ercolano, Elizabeth; Zhou, Yang; Sanft, Tara; Gross, Cary; Schmitz, Kathryn; Neogi, Tuhina; Hershman, Dawn; Ligibel, Jennifer; Irwin, Melinda L. | 2016 | Exercise adherence in a randomized trial of exercise on aromatase inhibitor arthralgias in breast cancer survivors: The Hormones and Physical Exercise (HOPE) Study | 2 |
| Armbruster, Shannon D.; Song, Jaejoon; Gatus, Leticia; Lu, Karen H.; Basen-Engquist, Karen M. | 2018 | Endometrial cancer survivors' sleep patterns before and after a physical activity intervention: A retrospective cohort analysis | 2 |
| Bade, Brett C.; Hyer, J. Madison; Bevill, Benjamin T.; Pastis, Alex; Rojewski, Alana M.; Toll, Benjamin A.; Silvestri, Gerard A | 2018 | A Patient-Centered Activity Regimen Improves Participation in Physical Activity Interventions in Advanced-Stage Lung Cancer | 1 |
| Blackburn, Roxann; Presson, Kimberly; Laufman, Robin; Tomczak, Nancy; Brassil, Kelly J. | 2016 | Establishing an Inpatient Gym for Recipients of Stem Cell Transplantation: A Multidisciplinary Collaborative | 4 |
| Blaney, Janine; Lowe-Strong, Andrea; Rankin, Jane; Campbell, Anna; Allen, James; Gracey, Jackie | 2010 | The Cancer Rehabilitation Journey: Barriers to and Facilitators of Exercise Among Patients With Cancer-Related Fatigue | 4 |
| Bolam K, Mijwel S, Rundqvist H, Wengstrom Y | 2019 | Two-year follow-up of the Opti-Train randomised controlled exercise trial | 2 |
| Bourke, Liam; Doll, Helen; Crank, Helen; Daley, Amanda; Rosario, Derek; Saxton, John M. | 2011 | Lifestyle intervention in men with advanced prostate cancer receiving androgen suppression therapy: a feasibility study | 2 |
| Bositis, A.; Scanlon, T. S.; Hall, P. S.; Mock, V. | 2004 | Increasing exercise adherence in a clinical trial evaluating the effectiveness of exercise on cancer treatment-related fatigue | 3 |
| Broderick, J. M.; Guinan, E.; Kennedy, M. J.; Hollywood, D.; Courneya, K. S.; Culos-Reed, S. N.; Bennett, K.; O' Donnell, D. M.; Hussey, J. | 2013 | Feasibility and efficacy of a supervised exercise intervention in de-conditioned cancer survivors during the early survivorship phase: the PEACH trial | 2 |
| Broderick, J. M.; Guinan, E.; O' Donnell, D. M.; Hussey, J.; Tyrrell, E.; Normand, C. | 2014 | Calculating the costs of an 8-week, physiotherapy-led exercise intervention in deconditioned cancer survivors in the early survivorship period (the PEACH trial) | 2 |
| Carmack Taylor, Cindy L.; Demoor, Carl; Smith, Murray A.; Dunn, Andrea L.; Basen-Engquist, Karen; Nielsen, Ingrid; Pettaway, Curtis; Sellin, Rena; Massey, Pamela; Gritz, Ellen R. | 2006 | Active for Life After Cancer: a randomized trial examining a lifestyle physical activity program for prostate cancer patients | 1 |
| Capozzi, Lauren C.; Boldt, Kevin R.; Lau, Harold; Shirt, Lisa; Bultz, Barry; Culos-Reed, S. Nicole | 2015 | A clinic-supported group exercise program for head and neck cancer survivors: managing cancer and treatment side effects to improve quality of life | 2 |
| Christensen, Jesper Frank; Simonsen, Casper; Hojman, Pernille | 2018 | Exercise Training in Cancer Control and Treatment | 2 |
| Colombo, Reyna; Doherty, Deborah; Seidell, Janet Wiechec; Linn, Stacy; Drouin, Jacqueline S | 2015 | Design, Implementation, and Sustainability of Physical Therapy in a Comprehensive Oncology Survivorship Program -- A Case Report | 1 |
| Cormie, Prue; Turner, Brooke; Kaczmarek, Elizabeth; Drake, Deirdre; Chambers, Suzanne K | 2015 | A Qualitative Exploration of the Experience of Men With Prostate Cancer Involved in Supervised Exercise Programs | 3 |
| Courneya, Kerry S.; Segal, Roanne J.; Mackey, John R.; Gelmon, Karen; Reid, Robert D.; Friedenreich, Christine M.; Ladha, Aliya B.; Proulx, Caroline; Vallance, Jeffrey K. H.; Lane, Kirstin; Yasui, Yutaka; McKenzie, Donald C. | 2007 | Effects of aerobic and resistance exercise in breast cancer patients receiving adjuvant chemotherapy: a multicenter randomized controlled trial | 3 |
| Courneya, Kerry S.; Segal, Roanne J.; Gelmon, Karen; Reid, Robert D.; Mackey, John R.; Friedenreich, Christine M.; Proulx, Caroline; Lane, Kirstin; Ladha, Aliya B.; Vallance, Jeffrey K.; McKenzie, Donald C. | 2008 | Predictors of Supervised Exercise Adherence during Breast Cancer Chemotherapy | 2 |
| Demark-Wahnefried, Wendy; Rogers, Laura Q.; Alfano, Catherine M.; Thomson, Cynthia A.; Courneya, Kerry S.; Meyerhardt, Jeffrey A.; Stout, Nicole L.; Kvale, Elizabeth; Ganzer, Heidi; Ligibel, Jennifer A. | 2015 | Practical clinical interventions for diet, physical activity, and weight control in cancer survivors | 3 |
| Demark-Wahnefried, Wendy; Schmitz, Kathryn H.; Alfano, Catherine M.; Bail, Jennifer R.; Goodwin, Pamela J.; Thomson, Cynthia A.; Bradley, Don W.; Courneya, Kerry S.; Befort, Christie A.; Denlinger, Crystal S.; Ligibel, Jennifer A.; Dietz, William H.; Stolley, Melinda R.; Irwin, Melinda L.; Bamman, Marcas M.; Apovian, Caroline M.; Pinto, Bernardine M.; Wolin, Kathleen Y.; Ballard, Rachel M.; Dannenberg, Andrew J.; Eakin, Elizabeth G.; Longjohn, Matt M.; Raffa, Susan D.; Adams-Campbell, Lucile L.; Buzaglo, Joanne S.; Nass, Sharyl J.; Massetti, Greta M.; Balogh, Erin P.; Kraft, Elizabeth S.; Parekh, Anand K.; Sanghavi, Darshak M.; Morris, G. Stephen; Basen-Engquist, Karen | 2018 | Weight management and physical activity throughout the cancer care continuum | 3 |
| Donnelly, C. M.; Blaney, J. M.; Lowe-Strong, A.; Rankin, J. P.; Campbell, A.; McCrum-Gardner, E.; Gracey, J. H | 2011 | A randomised controlled trial testing the feasibility and efficacy of a physical activity behavioural change intervention in managing fatigue with gynaecological cancer survivors | 2 |
| Eakin, Elizabeth G.; Hayes, Sandra C.; Haas, Marion R.; Reeves, Marina M.; Vardy, Janette L.; Boyle, Frances; Hiller, Janet E.; Mishra, Gita D.; Goode, Ana D.; Jefford, Michael; Koczwara, Bogda; Saunders, Christobel M.; Demark-Wahnefried, Wendy; Courneya, Kerry S.; Schmitz, Kathryn H.; Girgis, Afaf; White, Kate; Chapman, Kathy; Boltong, Anna G.; Lane, Katherine; McKiernan, Sandy; Millar, Lesley; O'Brien, Lorna; Sharplin, Greg; Baldwin, Polly; Robson, Erin L. | 2015 | Healthy Living after Cancer: a dissemination and implementation study evaluating a telephone-delivered healthy lifestyle program for cancer survivors | 2 |
| Eckert, Katharina; Lange, Martin; Huber, Gerhard | 2012 | Effects of supplemental behavior-oriented exercise intervention in a disease management program for breast cancer | 3 |
| Fong, A. J.; Jones, J. M.; Faulkner, G.; Sabiston, C. M | 2018 | Exploring cancer centres for physical activity and sedentary behaviour support for breast cancer survivors | 1 |
| Fong, Angela J.; Faulkner, Guy; Jones, Jennifer M.; Sabiston, Catherine M. | 2018 | A qualitative analysis of oncology clinicians' perceptions and barriers for physical activity counseling in breast cancer survivors | 1 |
| Foucaut, Aude-Marie; Morelle, Magali; Kempf-Lépine, Anne-Sophie; Baudinet, Cédric; Meyrand, Renaud; Guillemaut, Séverine; Metzger, Séverine; Bourne-Branchu, Valérie; Grinand, Elodie; Chabaud, Sylvie; Pérol, David; Carretier, Julien; Berthouze, Sophie E.; Reynes, Eric; Perrier, Lionel; Rebattu, Paul; Heudel, Pierre-Etienne; Bachelot, Thomas; Bachmann, Patrick; Fervers, Béatrice; Trédan, Olivier; Touillaud, Marina | 2019 | Feasibility of an exercise and nutritional intervention for weight management during adjuvant treatment for localized breast cancer: the PASAPAS randomized controlled trial | 2 |
| Fox, L.; Cahill, F.; Burgess, C.; Peat, N.; Rudman, S.; Kinsella, J.; Cahill, D.; George, G.; Santaolalla, A.; Van Hemelrijck, M. | 2017 | Real World Evidence: A Quantitative and Qualitative Glance at Participant Feedback from a Free-Response Survey Investigating Experiences of a Structured Exercise Intervention for Men with Prostate Cancer | 3 |
| Freitag, Nils; Weber, Pia Deborah; Sanders, Tanja Christiane; Schulz, Holger; Bloch, Wilhelm; Schumann, Moritz | 2018 | High-intensity interval training and hyperoxia during chemotherapy: A case report about the feasibility, safety and physical functioning in a colorectal cancer patient - | 2 |
| Galvão, Daniel A.; Newton, Robert U.; Girgis, Afaf; Lepore, Stephen J.; Stiller, Anna; Mihalopoulos, Cathrine; Gardiner, Robert A.; Taaffe, Dennis R.; Occhipinti, Stefano; Chambers, Suzanne K. | 2018 | Randomized controlled trial of a peer led multimodal intervention for men with prostate cancer to increase exercise participation | 1 |
| Gell, Nancy M.; Grover, Kristin W.; Humble, Morgan; Sexton, Michelle; Dittus, Kim | 2017 | Efficacy, feasibility, and acceptability of a novel technology-based intervention to support physical activity in cancer survivors | 2 |
| Grabenbauer, Alexander; Grabenbauer, Andrea J.; Lengenfelder, Rosa; Grabenbauer, Gerhard G.; Distel, Luitpold V. | 2016 | Feasibility of a 12-month-exercise intervention during and after radiation and chemotherapy in cancer patients: impact on quality of life, peak oxygen consumption, and body composition | 2 |
| Granger, Catherine L.; Denehy, Linda; Remedios, Louisa; Retica, Sarah; Phongpagdi, Pimsiri; Hart, Nicholas; Parry, Selina M. | 2016 | Barriers to Translation of Physical Activity into the Lung Cancer Model of Care. A Qualitative Study of Clinicians' Perspectives | 1 |
| Granger, Catherine L.; Parry, Selina M.; Denehy, Linda; Remedios, Louisa | 2018 | Evidence, education and multi-disciplinary integration are needed to embed exercise into lung cancer clinical care: A qualitative study involving physiotherapists | 1 |
| Greterman, Sarah Jane | 2018 | Changes in physical activity and quality of life of cancer survivors participating in a group exercise program | 2 |
| Groeneveldt, Lara; Mein, Gill; Garrod, Rachel; Jewell, Andrew P.; Van Someren, Ken; Stephens, Richard; D'Sa, Shirley P.; Yong, Kwee L | 2013 | A mixed exercise training programme is feasible and safe and may improve quality of life and muscle strength in multiple myeloma survivors | 2 |
| Haines, T. P.; Sinnamon, P.; Wetzig, N. G.; Lehman, M.; Walpole, E.; Pratt, T.; Smith, A. | 2010 | Multimodal exercise improves quality of life of women being treated for breast cancer, but at what cost? Randomized trial with economic evaluation | 2 |
| Haussmann, Alexander; Gabrian, Martina; Ungar, Nadine; Jooß, Stefan; Wiskemann, Joachim; Sieverding, Monika; Steindorf, Karen | 2018 | What hinders healthcare professionals in promoting physical activity towards cancer patients? The influencing role of healthcare professionals' concerns, perceived patient characteristics and perceived structural factors | 1 |
| Hayes, Sandra C.; Johansson, Karin; Alfano, Catherine M.; Schmitz, Kathryn | 2011 | Exercise for breast cancer survivors: bridging the gap between evidence and practice | 2 |
| Hayes, Sandra; Rye, Sheree; Battistutta, Diana; Yates, Patsy; Pyke, Chris; Bashford, John; Eakin, Elizabeth | 2011 | Design and implementation of the Exercise for Health trial -- a pragmatic exercise intervention for women with breast cancer | 3 |
| Hayes, Sandra C.; Rye, Sheree; Disipio, Tracey; Yates, Patsy; Bashford, John; Pyke, Chris; Saunders, Christobel; Battistutta, Diana; Eakin, Elizabeth | 2013 | Exercise for health: a randomized, controlled trial evaluating the impact of a pragmatic, translational exercise intervention on the quality of life, function and treatment-related side effects following breast cancer | 2 |
| Hoffman, Amy J.; Brintnall, Ruth Ann; Brown, Jean K.; von Eye, Alexander; Jones, Lee W.; Alderink, Gordon; Ritz-Holland, Debbie; Enter, Mark; Patzelt, Lawrence H.; VanOtteren, Glenn M. | 2013 | Too Sick Not to Exercise | 2 |
| Hoffman, Amy J.; Brintnall, Ruth Ann | 2017 | A Home-based Exercise Intervention for Non-Small Cell Lung Cancer Patients Post-Thoracotomy | 1 |
| Huether, Katie; Abbott, Linda; Cullen, Laura; Cullen, Liz; Gaarde, Ami | 2016 | Energy Through Motion©: An Evidence-Based Exercise Program to Reduce Cancer-Related Fatigue and Improve Quality of Life | 1 |
| Hubbard, Gill; Campbell, Anna; Fisher, Abi; Harvie, Michelle; Maltinsky, Wendy; Mullen, Russell; Banks, Elspeth; Gracey, Jackie; Gorely, Trish; Munro, Julie; Ozakinci, Gozde | 2018 | Physical activity referral to cardiac rehabilitation, leisure centre or telephone-delivered consultations in post-surgical people with breast cancer: a mixed methods process evaluation | 4 |
| Hubbard, Gill; Adams, Richard; Campbell, Anna; Kidd, Lisa; Leslie, Stephen J.; Munro, Julie; Watson, Angus | 2016 | Is referral of postsurgical colorectal cancer survivors to cardiac rehabilitation feasible and acceptable? A pragmatic pilot randomised controlled trial with embedded qualitative study | 2 |
| Irwin - British Journal of Sports Medicine | 2009 | Physical activity interventions for cancer survivors | 1 |
| Jahn, Patrick; Lakowa, Nicole; Landenberger, Margarete; Vordermark, Dirk; Stoll, Oliver | 2012 | InterACTIV: an exploratory study of the use of a game console to promote physical activation of hospitalized adult patients with cancer | 2 |
| Johansson, Karin; Hayes, Sandi; Speck, Rebecca M.; Schmitz, Kathryn H. | 2013 | Water-based exercise for patients with chronic arm lymphedema: a randomized controlled pilot trial | 3 |
| James‐Martin, G.; Koczwara, B.; Smith, E. L.; Miller, M. D. | 2014 | Information needs of cancer patients and survivors regarding diet, exercise and weight management: A qualitative study | 1 |
| Kampshoff, C, van Dongen J, va Mechelen W, Schep G, Vreugdenhil A, Twisk J, Bosman J, Brug J, Chinapaw M, Buffart L. | 2018 | Long-term effectiveness and cost-effectiveness of high versus low-to-moderate intensity resistance and endurance exercise interventions among cancer survivors | 2 |
| Kristiansen, Maria; Adamsen, Lis; Piil, Karin; Halvorsen, Ida; Nyholm, Nanna; Hendriksen, Carsten | 2017 | A three-year national follow-up study on the development of community-level cancer rehabilitation in Denmark | 1 |
| Knobf, M. Tish; Thompson, A. Siobhan; Fennie, Kristopher; Erdos, Diane | 2014 | The effect of a community-based exercise intervention on symptoms and quality of life | 2 |
| Kim, Soo Hyun; Shin, Mi Soon; Lee, Han Sul; Lee, Eun Sook; Ro, Jung Sil; Kang, Han Sung; Kim, Seok Won; Lee, Won Hee; Kim, Hee Soon; Kim, Chun Ja; Kim, Joohyung; Yun, Young Ho | 2011 | Randomized Pilot Test of a Simultaneous Stage-Matched Exercise and Diet Intervention for Breast Cancer Survivors | 2 |
| Kim, Sue; Ko, Yun Hee; Song, Yoonkyung; Kang, Min Jae; Lee, Hyojin; Kim, Sung Hae; Jeon, Justin Y.; Cho, Young Up; Yi, Gihong; Han, Jeehee | 2019 | Development of an exercise adherence program for breast cancer survivors with cancer-related fatigue-an intervention mapping approach | 3 |
| Kwiatkowski, Fabrice; Mouret-Reynier, Marie-Ange; Duclos, Martine; Bridon, François; Hanh, Thierry; Van Praagh-Doreau, Isabelle; Travade, Armelle; Vasson, Marie-Paule; Jouvency, Sylvie; Roques, Christian; Bignon, Yves-Jean | 2017 | Long-term improvement of breast cancer survivors' quality of life by a 2-week group physical and educational intervention: 5-year update of the 'PACThe' trial | 1 |
| Kuehr, L. E. A.; Wiskemann, Joachim; Abel, Ulrich; Ulrich, Cornelia M.; Hummler, Simone; Thomas, Michael | 2014 | Exercise in Patients with Non-Small Cell Lung Cancer | 2 |
| Ligibel, Jennifer A.; Jones, Lee W.; Brewster, Abenaa M.; Clinton, Steven K.; Korde, Larissa A.; Oeffinger, Kevin C.; Bender, Catherine M.; Tan, Winston; Merrill, Janette K.; Katta, Sweatha Alfano; Catherine M. | 2019 | Oncologists' Attitudes and Practice of Addressing Diet, Physical Activity, and Weight Management With Patients With Cancer: Findings of an ASCO Survey of the Oncology Workforce | 1 |
| Loh, Kah Poh; Kleckner, Ian R.; Lin, Po‐Ju; Mohile, Supriya G.; Canin, Beverly E.; Flannery, Marie A.; Fung, Chunkit; Dunne, Richard F.; Bautista, Javier; Culakova, Eva; Kleckner, Amber S.; Peppone, Luke J.; Janelsins, Michelle; McHugh, Colin; Conlin, Alison; Cho, Jonathan K.; Kasbari, Sameer; Esparaz, Benjamin T.; Kuebler, J. Philip; Mustian, Karen M. | 2019 | Effects of a Home‐based Exercise Program on Anxiety and Mood Disturbances in Older Adults with Cancer Receiving Chemotherapy | 2 |
| Lee, Morgan S.; Small, Brent J.; Jacobsen, Paul B. | 2017 | Rethinking barriers: a novel conceptualization of exercise barriers in cancer survivors | 1 |
| Macleod, Maureen; Steele, Robert J. C.; O'Carroll, Ronan E.; Wells, Mary; Campbell, Anna; Sugden, Jacqui A.; Rodger, Jackie; Stead, Martine; McKell, Jennifer; Anderson, Annie S. | 2018 | Feasibility study to assess the delivery of a lifestyle intervention (TreatWELL) for patients with colorectal cancer undergoing potentially curative treatment | 2 |
| Marthick, Michael; Grant, Suzanne J.; Lacey, Judith | 2019 | Establishing an integrative oncology service in the Australian healthcare setting-the Chris O'Brien Lifehouse Hospital experience | 1 |
| McIntosh, Megan; Opozda, Melissa; Galvão, Daniel A.; Chambers, Suzanne K.; Short, Camille E. | 2019 | Identifying the exercise-based support needs and exercise programme preferences among men with prostate cancer during active surveillance: A qualitative study | 3 |
| Matthews, Charles E.; Wilcox, Sara; Hanby, Cara L.; Der Ananian, Cheryl; Heiney, Sue P.; Gebretsadik, Tebeb; Shintani, Ayumi | 2007 | Evaluation of a 12-week home-based walking intervention for breast cancer survivors | 2 |
| Maxwell‐Smith, Chloe; Zeps, Nik; Hagger, Martin S.; Platell, Cameron; Hardcastle, Sarah J. | 2017 | Barriers to physical activity participation in colorectal cancer survivors at high risk of cardiovascular disease | 3 |
| Mayer, Deborah K.; Landucci, Gina; Awoyinka, Lola; Atwood, Amy K.; Carmack, Cindy L.; Demark-Wahnefried, Wendy; McTavish, Fiona; Gustafson, David H. | 2018 | SurvivorCHESS to increase physical activity in colon cancer survivors: can we get them moving? | 1 |
| Mewes, Janne C.; Steuten, Lotte M. G.; Ijsbrandy, Charlotte; Ijzerman, Maarten J.; van Harten, Wim H. | 2017 | Value of Implementation of Strategies to Increase the Adherence of Health Professionals and Cancer Survivors to Guideline-Based Physical Exercise | 4 |
| Midtgaard, J.; Røssell, K.; Christensen, J. F.; Uth, J.; Adamsen, L.; Rørth, M.; Midtgaard, Julie; Røssell, Kasper; Christensen, Jesper Frank; Uth, Jacob; Adamsen, Lis; Rørth, Mikael | 2012 | Demonstration and manifestation of self-determination and illness resistance--a qualitative study of long-term maintenance of physical activity in posttreatment cancer survivors | 3 |
| Mehnert, Anja; Veers, Silke; Howaldt, Dirk; Braumann, Klaus-Michael; Koch, Uwe; Schulz, Karl-Heinz | 2011 | Effects of a physical exercise rehabilitation group program on anxiety, depression, body image, and health-related quality of life among breast cancer patients | 2 |
| Musanti, Rita; Ying-Yu, Chao; Collins, Katelyn | 2019 | Fitness and Quality of Life Outcomes of Cancer Survivor Participants in a Community Exercise Program | 3 |
| Nadler, Michelle B.; Bainbridge, Daryl; Fong, Angela J.; Sussman, Jonathan; Tomasone, Jennifer R.; Neil-Sztramko, Sarah E. | 2019 | Moving Cancer Care Ontario's Exercise for People with Cancer guidelines into oncology practice: using the Theoretical Domains Framework to validate a questionnaire | 3 |
| Nock, Nora L.; Owusu, Cynthia; Kullman, Emily L.; Austin, Kris; Roth, Beth; Cerne, Stephen; Harmon, Carl; Moore, Halle; Vargo, Mary; Hergenroeder, Paul; Malone, Hermione; Rocco, Michael; Tracy, Russell; Lazarus, Hillard M.; Kirwan, John P.; Heyman, Ellen; Berger, Nathan A. | 2013 | A Community-Based Exercise and Support Group Program in African-American Breast Cancer Survivors (ABCs) | 2 |
| Olivier, Cecile; Grosbois, Jean-Marie; Cortot, Alexis B.; Peres, Sophie; Heron, Christophe; Delourme, Julie; Gierczynski, Marianne; Hoorelbeke, Anne; Scherpereel, Arnaud; Le Rouzic, Olivier | 2018 | Real-life feasibility of home-based pulmonary rehabilitation in chemotherapy-treated patients with thoracic cancers: a pilot study | 2 |
| Oertle, Staci; Burrell, Sherry; Pirollo, Melanie | 2016 | Evaluating the Effects of a Physician-Referred Exercise Program on Cancer-Related Fatigue and Quality of Life Among Early Cancer Survivors | 2 |
| Ottenbacher, A. J.; Day, R. S.; Taylor, W. C.; Sharma, S. V.; Sloane, R.; Snyder, D. C.; Lipkus, I. M.; Jones, L. W.; Demark-Wahnefried, W.; Ottenbacher, Allison J.; Day, R. Sue; Taylor, Wendell C.; Sharma, Shreela V.; Sloane, Richard; Snyder, Denise C.; Lipkus, Isaac M.; Jones, Lee W.; Demark-Wahnefried, Wendy | 2012 | Long-term physical activity outcomes of home-based lifestyle interventions among breast and prostate cancer survivors | 1 |
| Pullen, Tanya; Sharp, Paul; Bottorff, Joan L.; Sabiston, Catherine M.; Campbell, Kristin L.; Ellard, Susan L.; Gotay, Carolyn; Fitzpatrick, Kayla; Caperchione, Cristina M. | 2018 | Acceptability and satisfaction of project MOVE: A pragmatic feasibility trial aimed at increasing physical activity in female breast cancer survivors | 4 |
| Pablo, Susana; Arietaleanizbeaskoa, Maria Soledad; Mendizabal, Nere; Luis, Raquel;  Gil, Erreka; de la Fuente, Ibon; Rogers, Heather;  Grandes, Gonzalo | 2019 | Linkages between health and community organizations for increasing longterm adherence to physical exercise: experiences of patients involved in the EfiKroniK Program | 2 |
| Parker, Nathan H.; Lee, Rebecca E.; O'Connor, Daniel P.; An, Ngo-Huang; Petzel, Maria Q. B.; Schadler, Keri; Xuemei, Wang; Lianchun, Xiao; Fogelman, David; Simpson, Richard; Fleming, Jason B.; Lee, Jeffrey E.; Ching-Wei, D. Tzeng; Sahai, Sunil K.; Basen-Engquist, Karen; Katz, Matthew H. G. | 2019 | Supports and Barriers to Home-Based Physical Activity During Preoperative Treatment of Pancreatic Cancer: A Mixed-Methods Study | 3 |
| Peddle‐McIntyre, C. J.; Baker, M. K.; Lee, Y. C. G.; Galvão, D. A.; Cormie, P.; Graham, V.; Newton, R. U. | 2018 | The feasibility of a pragmatic distance‐based intervention to increase physical activity in lung cancer survivors | 2 |
| Peeters, C.; Stewart, A.; Segal, R.; Wouterloot, E.; Scott, C. G.; Aubry, T. | 2009 | Evaluation of a cancer exercise program: patient and physician beliefs | 1 |
| Perrier, Lionel; Foucaut, Aude-Marie; Morelle, Magali; Touillaud, Marina; Kempf-Lépine, Anne-Sophie; Heinz, Dominik; Gomez, Frédéric; Meyrand, Renaud; Baudinet, Cédric; Berthouze, Sophie; Reynes, Eric; Carretier, Julien; Guillemaut, Séverine; Pérol, David; Trédan, Olivier; Philip, Thierry; Bachmann, Patrick;  Fervers, Béatrice | 2019 | Cost-effectiveness of an exercise and nutritional intervention versus usual nutritional care during adjuvant treatment for localized breast cancer: the PASAPAS randomized controlled trial | 3 |
| Persoon, S.; Chinapaw, M. J. M.; Buffart, L. M.; Brug, J.; Kersten, M. J.; Nollet, F. | 2018 | Lessons learnt from a process evaluation of an exercise intervention in patients treated with autologous stem cell transplantation | 4 |
| Petersson, Lena-Marie; Berglund, Gunilla; Brodin, Ola; Glimelius, Bengt; Sjödén, Per-Olow | 2000 | Group rehabilitation for cancer patients: Satisfaction and perceived benefits | 1 |
| Phillips, Siobhan M.; Courneya, Kerry S.; Welch, Whitney A.; Gavin, Kara L.; Cottrell, Alison; Nielsen, Anne; Solk, Payton; Blanch-Hartigan, Danielle; Cella, David; Ackermann, Ronald T.; Spring, Bonnie; Penedo, Frank | 2019 | Breast cancer survivors' preferences for mHealth physical activity interventions: findings from a mixed methods study | 1 |
| Pinto, B. M.; Rabin, C.; Dunsiger, S.; Pinto, Bernardine M.; Rabin, Carolyn; Dunsiger, Shira | 2009 | Home-based exercise among cancer survivors: adherence and its predictors | 2 |
| Pinto, Bernardine; Waldemore, Marissa; Rosen, Rochelle | 2015 | A Community-Based Partnership to Promote Exercise Among Cancer Survivors: Lessons Learned | 1 |
| Plumeau, K.; Marcyan, R. A.; Heubeck, A.; Thomas, Z.; Fitzsimons, J.; Mayer, J. E. | 2019 | Home exercise programs for cancer survivors: can a weekly phone call impact compliance, function or quality of life? | 2 |
| Predeger, Elizabeth J.; O'Malley, Maureen; Hendrix, Thomas; Parker, Nadine M. | 2014 | Oncology rehabilitation outcomes over time: a mixed-methods approach | 3 |
| Pullen, Tanya; Bottorff, Joan L.; Sabiston, Catherine M.; Campbell, Kristin L.; Eves, Neil D.; Ellard, Susan L.; Gotay, Carolyn; Fitzpatrick, Kayla; Sharp, Paul; Caperchione, Cristina M. | 2018 | Utilizing RE-AIM to examine the translational potential of Project MOVE, a novel intervention for increasing physical activity levels in breast cancer survivors | 4 |
| Rabin, Carolyn; Pinto, Bernardine; Fava, Joseph | 2016 | Randomized trial of a physical activity and meditation intervention for young adult cancer survivors | 2 |
| Retèl, Valesca P.; van der Molen, Lisette; Hilgers, Frans J. M.; Rasch, Coen R. N.; L'Ortye, Annemiek A. A. M. H. J.; Steuten, Lotte M. G.; van Harten, Wim H. | 2011 | A cost-effectiveness analysis of a preventive exercise program for patients with advanced head and neck cancer treated with concomitant chemo-radiotherapy | 3 |
| Robertson, Lindsay; Richards, Rosalina; Egan, Richard; Szymlek‐Gay, Ewa A. | 2013 | Promotion and support of physical activity among cancer survivors: A service provider perspective | 1 |
| Rogers, Laura Q.; Vicari, Sandy; Courneya, Kerry S. | 2010 | Lessons learned in the trenches: facilitating exercise adherence among breast cancer survivors in a group setting | 3 |
| Reynolds, Jana; Thibodeaux, Lorie; Jiang, Luohua; Francis, Kevin; Hochhalter, Angie | 2015 | Fit & strong! Promotes physical activity and well-being in older cancer survivors | 2 |
| Roagenhofer, S.; Wortz, I.; Widmann, T. | 2015 | Interim analysis of the SENSe-study structured evaluation of sustainability of sports after cancer | 5 |
| Roggenhofer, S.; Widmann, T. | 2016 | SENSe-Study interim analysis structured evaluation of sustainability of sports after cancer | 5 |
| Sabiston, Catherine M.; Fong, Angela J.; O'Loughlin, Erin K.; Meterissian, Sarkis | 2019 | A mixed-methods evaluation of a community physical activity program for breast cancer survivors | 2 |
| Santa Mina D, Sabiston C, Au D, Fong A, Capozzi L, Langelier D, Chasen M, Chiarotto J, Tomasone J, Jones J, Chang E, Culos-Reed S. | 2018 | Connecting people with cancer to physical activity and exercise programs: a pathway to create accessibility and engagement | 1 |
| Scaramuzzo, Leah A.; Gordils-Perez, Janet; Cullen, Patsy McGuire | 2014 | Getting patients active: using national data to drive practice | 1 |
| Schmidt, Thorsten; Schwarz, Madalena; Van Mackelenbergh, Marion; Jonat, Walter; Weisser, Burkhard; Röcken, Christoph; Mundhenke, Christoph | 2017 | Feasibility study to evaluate compliance of physical activity over a long time period and its influence on the total activity score, glucose metabolism and physical and psychological parameters following breast cancer | 2 |
| Schmitz, Kathryn H. | 2017 | Incorporating strength training into cancer care: Translating pal into the strength after breast cancer program | 1 |
| Schmitz, Kathryn H. | 2011 | Exercise for secondary prevention of breast cancer: moving from evidence to changing clinical practice | 1 |
| Schmitz, Kathryn H.; Troxel, Andrea B.; Dean, Lorraine T.; DeMichele, Angela; Brown, Justin C.;  Sturgeon, Kathleen; Zhang, Zi; Evangelisti, Margaret; Spinelli, Bryan; Kallan, Michael J.; Denlinger, Crystal; Cheville, Andrea; Winkels, Renate M.; Chodosh, Lewis; Sarwer, David B. | 2019 | Effect of Home-Based Exercise and Weight Loss Programs on Breast Cancer–Related Lymphedema Outcomes Among Overweight Breast Cancer Survivors: The WISER Survivor Randomized Clinical Trial | 2 |
| Schmitz, Kathryn H.; Campbell, Anna M.; Stuiver, Martijn M.; Pinto, Bernardine M.; Schwartz, Anna L.; Morris, G. Stephen; Ligibel, Jennifer A.;  Cheville, Andrea; Galvão, Daniel A.; Alfano, Catherine M.; Patel, Alpa V.; Hue, Trisha; Gerber, Lynn H.; Sallis, Robert; Gusani, Niraj J.; Stout, Nicole L.; Chan, Leighton; Flowers, Fiona; Doyle, Colleen; Helmrich, Susan; Bain, William; Sokolof, Jonas; Winters-Stone, Kerri M.; Campbell, Kristin L.; Matthews, Charles E. | 2019 | Exercise is medicine in oncology: Engaging clinicians to help patients move through cancer | 1 |
| Schneider, C. M.; Dennehy, C. A.; Roozeboom, M.; Carter, S. D. | 2002 | A model program: exercise intervention for cancer rehabilitation | 3 |
| Schumacher, Molly M.; McNiel, Paula | 2018 | The Impact of Livestrong® at the YMCA for Cancer Survivors | 3 |
| Scott, Jessica M.; Iyengar, Neil M.; Nilsen, Tormod S.; Michalski, Meghan; Thomas, Samantha M.; Herndon, James, 2nd; Sasso, John; Yu, Anthony; Chandarlapaty, Sarat; Dang, Chau T.; Comen, Elizabeth A.; Dickler, Maura N.; Peppercorn, Jeffrey M.; Jones, Lee W. | 2018 | Feasibility, safety, and efficacy of aerobic training in pretreated patients with metastatic breast cancer: A randomized controlled trial | 2 |
| Sheehan, P.; Denieffe, S.; Harrison, M. | 2016 | Evaluation of a Sustainable Intervention using Exercise - for Cancer Fatigue (ESIE-CF Trial) | 2 |
| Sheill, G.; Guinan, E.; Neill, L. O.; Hevey, D.; Hussey, J. | 2018 | Physical activity and advanced cancer: the views of oncology and palliative care physicians in Ireland | 1 |
| Sheppard, Vanessa B.; Hicks, Jennifer; Makambi, Kepher; Hurtado-de-Mendoza, Alejandra; Demark-Wahnefried, Wendy; Adams-Campbell, Lucile | 2016 | The feasibility and acceptability of a diet and exercise trial in overweight and obese black breast cancer survivors: The Stepping STONE study | 2 |
| Short, Camille E.; James, Erica L.; Girgis, Afaf; D'Souza, Mario I.; Plotnikoff, Ronald C. | 2015 | Main outcomes of the Move More for Life Trial: a randomised controlled trial examining the effects of tailored-print and targeted-print materials for promoting physical activity among post-treatment breast cancer survivors | 1 |
| Short, Camille E.; James, Erica L.; Plotnikoff, Ronald C. | 2013 | Theory-and evidence-based development and process evaluation of the Move More for Life program: a tailored-print intervention designed to promote physical activity among post-treatment breast cancer survivors | 3 |
| Siedentopf, F.; Utz-Billing, I.; Gairing, S.; Schoenegg, W.; Kentenich, H.; Kollak, I. | 2013 | Yoga for Patients with Early Breast Cancer and its Impact on Quality of Life - a Randomized Controlled Trial | 3 |
| Smith-Turchyn, Jenna; Richardson, Julie; Tozer, Richard; McNeely, Margaret; Thabane, Lehana | 2019 | Bridging the gap: incorporating exercise evidence into clinical practice in breast cancer care | 2 |
| Spahn, Günther; Choi, Kyung-Eun; Ke | 2013 | Can a multimodal mind-body program enhance the treatment effects of physical activity in breast cancer survivors with chronic tumor-associated fatigue? A randomized controlled trial | 2 |
| Sprod, Lisa K.; Hsieh, City C.; Hayward, Reid; Schneider, Carole M. | 2010 | Three versus six months of exercise training in breast cancer survivors | 2 |
| Sremanakova, J.; Jones, D.; Cooke, R.; Burden, S. | 2019 | Exploring Views of Healthcare Professionals, Researchers, and People Living with and beyond Colorectal Cancer on a Healthy-Eating and Active Lifestyle Resource | 1 |
| Stacey, F. G.; James, E. L.; Chapman, K.; Lubans, D. R. | 2016 | Social cognitive theory mediators of physical activity in a lifestyle program for cancer survivors and carers: findings from the ENRICH randomized controlled trial | 4 |
| Stevinson, C.; Fox, K. R. | 2005 | Role of exercise for cancer rehabilitation in UK hospitals: a survey of oncology nurses | 1 |
| Stuecher, Katrin; Vogt, Lutz; Niederer, Daniel; Banzer, Winfried; Schmidt, Katharina; Bolling, Claus; Dignaß, Axel | 2019 | Exercise improves functional capacity and lean body mass in patients with gastrointestinal cancer during chemotherapy: a single-blind RCT | 2 |
| Sutton, Eileen; Hackshaw-McGeagh, Lucy; Aning, Jonathan; Bahl, Amit; Koupparis, Anthony; Persad, Raj; Martin, Richard; Lane, J.; Hackshaw-McGeagh, Lucy E.; Martin, Richard M.; Lane, J. Athene | 2017 | The provision of dietary and physical activity advice for men diagnosed with prostate cancer: a qualitative study of the experiences and views of health care professionals, patients and partners | 1 |
| Talbot Rice, Helena; Malcolm, Lorna; Norman, Kate; Jones, Alison; Lee, Katherine; Preston, Gail; McKenzie, David; Maddocks, Matthew | 2014 | An evaluation of the St Christopher's Hospice rehabilitation gym circuits classes: Patient uptake, outcomes, and feedback | 4 |
| Thorsen, Lene; Skovlund, Eva; Strømme, Sigmund B.; Hornslien, Kjersti; Dahl, Alv A.; Fosså, Sophie D. | 2005 | Effectiveness of physical activity on cardiorespiratory fitness and health-related quality of life in young and middle-aged cancer patients shortly after chemotherapy | 2 |
| Truong, Pauline T.; Gaul, Catherine A.; McDonald, Rachel E.; Petersen, Ross B.; Jones, Stuart O.; Alexander, Abraham S.; Lim, Jan T. W.; Ludgate, Charles | 2011 | Prospective evaluation of a 12-week walking exercise program and its effect on fatigue in prostate cancer patients undergoing radical external beam radiotherapy | 2 |
| Tsianakas, Vicki; Harris, Jenny; Ream, Emma; Van Hemelrijck, Mieke; Purushotham, Arnie; Mucci, Lorelei; Green, James S. A.; Fewster, Jacquetta; Armes, Jo | 2017 | CanWalk: a feasibility study with embedded randomised controlled trial pilot of a walking intervention for people with recurrent or metastatic cancer | 4 |
| Veal, I.; Peat, N.; Jones, G. D.; Tsianakas, V.; Armes, J. | 2019 | Missed opportunities for physical activity management at key points throughout the chemotherapy pathway for colorectal survivors: an observational interview study | 1 |
| Young-McCaughan, Stacey; Mays, Mary Z.; Arzola, Sonya M.; Yoder, Linda H.; Dramiga, Stacey A.; Leclerc, Kenneth M.; Caton, John R.; Sheffler, Robert L.; Nowlin, Marilyn U. | 2003 | Research and commentary: Change in exercise tolerance, activity and sleep patterns, and quality of life in patients with cancer participating in a structured exercise program | 3 |
| Young-McCaughan, S.; Mays, M. Z.; Arzola, S. M.; Yoder, L. H.; Dramiga, S. A.; Leclerc, K. M.; Caton, J. R., Jr.; Sheffler, R. L.; Nowlin, M. U. | 2003 | Change in exercise tolerance, activity and sleep patterns, and quality of life in patients with cancer participating in a structured exercise program...including commentary by Mock V | 3 |
| Young Ho, Yun; Young Ae, Kim; Myung Kyung, Lee; Jin Ah, Sim; Byung-Ho, Nam; Sohee, Kim; Eun Sook, Lee; Dong-Young, Noh; Jae-Young, Lim; Sung, Kim; Si-Young, Kim; Chi-Heum, Cho; Kyung Hae, Jung; Mison, Chun; Soon Nam, Lee; Kyong Hwa, Park; Sohee, Park; Yun, Young Ho; Kim, Young Ae; Lee, Myung Kyung | 2017 | A randomized controlled trial of physical activity, dietary habit, and distress management with the Leadership and Coaching for Health (LEACH) program for disease-free cancer survivors | 2 |
| Ying, Wang; Min, Qiang Wan; Lei, Tang; Na, Zheng Xiao; Li, Li; Jing, Li | 2019 | The health effects of Baduanjin exercise (a type of Qigong exercise) in breast cancer survivors: A randomized, controlled, single-blinded trial | 2 |
| Yang, Tsui-Yun; Chen, Mei-Ling; Li, Chia-Chun | 2015 | Effects of an aerobic exercise programme on fatigue for patients with breast cancer undergoing radiotherapy | 2 |
| Yochem, A.; Adams, B. E.; Minick, K.; Brennan, G. P. | 2018 | A standardized physical therapy cancer rehabilitation protocol in acute care for patients with hematological malignancies | 2 |
| Wonders, Karen Y.; Wise, Rob; Ondreka, Danielle; Gratsch, Josh | 2019 | Cost Savings Analysis of Individualized Exercise Oncology Programs | 3 |
| White, S. M.; McAuley, E.; Estabrooks, P. A.; Courneya, K. S.; White, Siobhan M.; McAuley, Edward; Estabrooks, Paul A.; Courneya, Kerry S. | 2009 | Translating physical activity interventions for breast cancer survivors into practice: an evaluation of randomized controlled trials | 1 |
| Webb, J.; Peel, J.; Fife-Schaw, C.; Ogden, J. | 2019 | A mixed methods process evaluation of a print-based intervention supported by internet tools to improve physical activity in UK cancer survivors | 1 |
| Webb, J.; Fife-Schaw, C.; Ogden, J. | 2019 | A randomised control trial and cost-consequence analysis to examine the effects of a print-based intervention supported by internet tools on the physical activity of UK cancer survivors | 1 |
| Wenzel, Jennifer A.; Griffith, Kathleen A.; Shang, Jingjing; Thompson, Carol B.; Hedlin, Haley; Stewart, Kerry J.; DeWeese, Theodore; Mock, Victoria | 2013 | Impact of a home-based walking intervention on outcomes of sleep quality, emotional distress, and fatigue in patients undergoing treatment for solid tumors | 2 |
| Wang, Ya-Jung | 2010 | Effects of a six-week home-based walking program on Taiwanese women newly diagnosed with early stage breast cancer | 2 |
| Vanlemmens, L.; Anota, A.; Bogart, E.; Nerich, V.; Cauchois, D.; Dewitte, A.; Dormeuil, E.; Lartigau, E.; Le Gall, F.; Mocaer, H.; et al., | 2019 | eMouvoir: randomized study estimating the impact of a personalized and remote support centered on physical activity (PA) for patients (pts) after breast cancer (BC) | 2 |
| van der Leeden, Marike; Balland, Chloé; Geleijn, Edwin; Huijsmans, Rosalie J.; Dekker, Joost; Paul, Marinus A.; Dickhoff, Chris; Stuiver, Martijn M. | 2019 | In-hospital mobilization, physical fitness and physical functioning following lung cancer surgery | 3 |
| van, Rooijen Stefanus; Molenaar, Charlotte J. L.; Schep, Goof; van Lieshout, Rianne; Beijer, Sandra; Dubbers, Rosalie; Rademakers, Nicky;  Papen-Botterhuis; Nicole E.; van Kempen Suzanne; Carli, Francesco; Roumen, Rudi;  Slooter, Gerrit. | 2019 | Making Patients Fit for Surgery: Introducing a Four Pillar Multimodal Prehabilitation Program in Colorectal Cancer | 2 |
| Veal, I; Peat, N.; Jones, G. D.; Tsianakas, V.; Armes, J. | 2019 | Missed opportunities for physical activity management at key points throughout the chemotherapy pathway for colorectal survivors: an observational interview study | 1 |
| von Gruenigen, Vivian; Frasure, Heidi; Kavanagh, Mary Beth; Janata, Jeffrey; Waggoner, Steven; Rose, Peter; Lerner, Edith; Courneya, Kerry S. | 2012 | Survivors of uterine cancer empowered by exercise and healthy diet (SUCCEED): a randomized controlled trial | 2 |
| Umstattd Meyer, M. Renée; Meyer, Andrew R.; Wu, Cindy; Bernhart, John | 2018 | When helping helps: exploring health benefits of cancer survivors participating in for-cause physical activity events | 3 |
| Ungar, Nadine; Tsiouris, Angeliki; Haussmann, Alexander; Herbolsheimer, Florian; Wiskemann, Joachim; Steindorf, Karen; Sieverding, Monika | 2019 | o rest or not to rest-Health care professionals' attitude toward recommending physical activity to their cancer patients | 1 |
| Vallance, Jeff; Lesniak, Susanne L.; Belanger, Lisa J.; Courneya, Kerry S. | 2010 | Development and assessment of a physical activity guidebook for the Colon Health and Life-Long Exercise Change (CHALLENGE) trial (NCIC CO.21) | 1 |
| Vallance, Jeffrey Kelcey Hayes | 2008 | Promoting physical activity in breast cancer survivors: A randomized controlled trial | 2 |
| Vallance, Jeff K.; Nguyen, Nga H.; Moore, Melissa M.; Reeves, Marina M.; Rosenberg, Dori E.; Boyle, Terry; Milton, Shakira; Friedenreich, Christine M.; English, Dallas R.; Lynch, Brigid M. | 2019 | Effects of the activity and technology (activate) intervention on health‐related quality of life and fatigue outcomes in breast cancer survivors | 1 |
| van Gemert, Willemijn A. M.; van der Palen, Job; Monninkhof, Evelyn M.; Rozeboom, Anouk; Peters, Roelof; Wittink, Harriet; Schuit, Albertine J.; Peeters, Petra H. | 2015 | Quality of Life after Diet or Exercise-Induced Weight Loss in Overweight to Obese Postmenopausal Women: The SHAPE-2 Randomised Controlled Trial | 3 |
| van Waart, Hanna; Stuiver, Martijn M.; van Harten, Wim H.; Geleijn, Edwin; Kieffer, Jacobien M.; Buffart, Laurien M.; de Maaker-Berkhof, Marianne; Boven, Epie; Schrama, Jolanda; Geenen, Maud M.; Meerum Terwogt, Jetske M.; van Bochove, Aart; Lustig, Vera; van den Heiligenberg, Simone M.; Smorenburg, Carolien H.; Hellendoorn-van Vreeswijk, Jeannette A. J. H.; Sonke, Gabe S.; Aaronson, Neil K. | 2015 | Effect of Low-Intensity Physical Activity and Moderate- to High-Intensity Physical Exercise During Adjuvant Chemotherapy on Physical Fitness, Fatigue, and Chemotherapy Completion Rates: Results of the PACES Randomized Clinical Trial | 2 |
| van Waart, Hanna; van Dongen, Johanna M.; van Harten, Wim H.; Stuiver, Martijn M.; Huijsmans, Rosalie; Hellendoorn-van Vreeswijk, Jeannette A. J. H.; Sonke, Gabe S.; Aaronson, Neil K | 2018 | Cost-utility and cost-effectiveness of physical exercise during adjuvant chemotherapy | 2 |
| Zopf, Eva M.; Bloch, W+A127:D134ilhelm; Machtens, Stefan; Zumbé, Jürgen; Rübben, Herbert; Marschner, Stefan; Kleinhorst, Christian; Schulte-Frei, Birgit; Herich, Lena; Felsch, Moritz; Predel, Hans-Georg; Braun, Moritz; Baumann, Freerk T. | 2015 | Effects of a 15-Month Supervised Exercise Program on Physical and Psychological Outcomes in Prostate Cancer Patients Following Prostatectomy: The ProRehab Study | 3 |

| 1 = no intervention (not an active intervention) |
| --- |
| 2 = study design excluded (efficacy) |
| 3 = wrong outcomes excluded (e.g. clinical outcomes) |
| 4 = patient population excluded (include palliative or under 18 years) |
| 5 = unable to source |
