# Supplementary material for: Exercise interventions for people diagnosed with cancer: a systematic review of implementation outcomes
Source: BMC Cancer. 2021 May 30;21:643. doi: 10.1186/s12885-021-08196-7 (PMC8166065; doi:10.1186/s12885-021-08196-7)
Supplement: Supplementary file 5 — Additional file 5: Supplementary Table 5. Summary of results Implementation Outcomes. [file 12885_2021_8196_MOESM5_ESM.docx]

**Supplementary Table 5: Summary of results Implementation Outcomes**

| **Acceptability** | | | | |
| --- | --- | --- | --- | --- |
| **Review** | **Year** | **Assessment tool/metric** | **Main Findings** | **Synthesis** |
| Kirkham | 2018 | Survey | Overall satisfaction was high. Positive results were reported for ease of attending sessions, clear program expectations, feeling supported, belief that they could continue with lifestyle changes and would recommend the program to others | 90.5% (n=5) acceptability; satisfaction rated as high |
| Leach | 2015 | Self-reported | 96.2% enjoyed the program and 89.8% looked forward to attending exercise sessions |  |
| Muraca | 2011 | Survey | 83.3% rated the program as valuable and useful |  |
| Rajotte | 2012 | Survey | Satisfaction with the classes was high, with all evaluation components above 95% |  |
| Santa Mina | 2019 | Survey | General program satisfaction was high: 85% of respondents strongly agreed with statements of enjoyment,  support of healthy behaviours, increased ability for activities of daily living, comfort with asking questions, and appropriateness of the exercise prescription |  |
| Sherman | 2010 | Survey | Overall satisfaction was high. Mean satisfaction rating of 28.16 (SD = 3.86) (maximum score 30) (94%) |  |
| **Adoption** | | | | |
| Beidas | 2014 | Barriers and enablers | *Barriers* - Variability in program costs covered by insurance, group-based structure difficult with patient population, cost to patients, labour intensive and confusing referral process, providers ability to determine patient eligibility *Enablers* - Established champion role, adapting the program (which included training staff to help with individualising programs, establishing a staff liaison role to co-ordinate referrals and calling patient who had been referred to confirm attendance at pre-program assessment. This phone call increases the portion of women who undertook the pre-program assessment from 39% to 65%) | Barriers - Cost (programs not subsided, implementation cost and costs to participants), low or cumbersome process for referrals, competing time demands of staff, lack of community resources to support ongoing activity, individual stage of change  Enablers - Champion, staff training to support individualised programming, dedicated role to optimising referrals, accessible location, simplifying assessment processes, referrals and delivery, ongoing evaluation during implementation to address administrative barriers to participation, community partnerships, using evidence to support program buy-in, extensive education resources |
| Bultijnck | 2018 | Organisation adoption + barriers and enablers | 65% of hospitals in Belgium had adopted exercise rehabilitation programs for cancer  *Barriers* - Low referral rate (19%) |  |
| Dalzell | 2017 | Barriers and enablers | *Barriers* - Competing demands of staff, lack of community resources to support transition to self-management, bottlenecks in the referral process *Enablers* - Interaction between medical team and program staff having access to medical charts |  |
| Dennett | 2017 | Healthcare provider barriers and enablers | *Barriers* - Referral process (particularly when clinicians are not co-located at the same site), insurance coverage of patients, location (car parking and travel distance), timing of programs, funding for equipment and advertising *Enablers* - Utilising multi-disciplinary services within other departments and in the community to meet complex patient needs, clinicians advocating for the program |  |
| Haas | 2011 | Organisation adoption | 14 sites have adopted FitSTEPS For Life across Dallas and Texas |  |
| Heston | 2015 | Organisation adoption | 416 sites have adopted LiveStrong YMCA across America |  |
| Irwin | 2017 | Organisation adoption | 18% of YMCA have adopted LiveStrong at the YMCA |  |
| Kimmel | 2014 | Barriers and enablers | *Enablers* - Accessible location (not gymnasium), establish a role to optimise referrals and negate the need for clinicians to explain program/benefits of exercise |  |
| Leach | 2014 | Barriers and enablers | *Barriers* - sourcing funding, oncologist time to discuss referral with potential patients *Enablers* - support from non-for-profit, cooperation and collaboration with local doctors, hospitals, cancer centres and other community organisations |  |
| Rogers | 2019 | Barriers and enablers | *Barriers*  Cost – participant costs (transportation costs, equipment (including clothing, childcare, gym fees) and other implementation costs (i.e. trainer certification, collecting and managing program data)  Stage of change – mental health as a barrier to being active  *Enablers*  Engagement - communication between cancer survivors and leadership to encourage and educate participants and local community, healthcare provider support and referrals, leveraging individuals who champion the program, building community partnership, enhancing  buy-in via communicating program benefits  Reflecting and evaluating – documenting health measures as a record implementation quality  Design quality and packaging - creating a program that encouraged support among participants  Cost - offsetting costs through charitable donations, grants and fundraisers  Evidence strength and quality - using research and participant testimonials to motivate participants and convince stakeholders of program benefits  Adaptability – programs should be adaptable (tailored) to patient need, including scheduling options and type of activity  Complexity – make the program easy to enrol in and implement. Make it initially easy for the patient to master exercise  Implementation readiness – educational resources (training modules, cheat sheets, intervention delivery scripts), practical cancer-specific training and observation of others to support delivery staff. Further, proper funding, staffing, training resources, telephone and community engagement were identified as important during implementation. Organisational resourcing important during program initiation but sustaining the program would require outside support (philanthropic)  Implementation climate - informing the community (especially cancer survivors) about the importance of exercise to facilitate program acceptance  Structural characteristics – the organisation paying for patients and transportation to their doctors  Patient need and resourcing – be cognisant of childcare needs, lack of exercise and nutrition knowledge, costs, socioeconomic status, and cancer-specific exercise modifications  Cosmopolitan - connecting with local retailers, religious organizations, fitness centres, rehabilitation facilities in order to provide supplies, funding, facilities and other types of support  Knowledge and beliefs – address declines in participant motivation through accountability and social support |  |
| Santa Mina | 2012 | Barriers and enablers | *Enablers* - accessible location, collegial support |  |
| Santa Mina | 2019 | Barriers and enablers | *Enablers:* reduced volume of clinical questionnaires, changed healthcare professional undertaking initial assessment to reduce duplication for patients. Ongoing evaluation during implementation resulted in addressing numerous administrative and logistical barriers that increased participation between pilot and roll-out from 44% to 83%, respectively |  |
| Sherman | 2010 | Organisation adoption | YWCA Encore delivered across 40 metropolitan and regional sites in Australia |  |
| Wurz | 2013 | Barriers and enablers | *Enablers* - training of instructors and resources (DVD (for patients)), evidence-based manual (for staff)), support from the local community and continuous program evaluation, tailoring exercise |  |
| **Appropriateness** | | | | |
| **Review** | **Year** | **Assessment tool/metric** |  | **Synthesis** |
| Beidas | 2014 | Previous efficacy study completed | The intervention was revised with oncology clinicians, physical therapists and survivors to improve the feasibility in practice. The modifications to the program were documented |  |
| Bjerre | 2018 | Previous efficacy study completed | Previous completion of small-scale explanatory randomized controlled trial demonstrated promising findings. Based on these findings, this study examined the real-world effectiveness of the intervention when delivered using pre-existing infrastructure | Most studies demonstrated appropriateness of exercise intervention through building upon a preliminary efficacy study in the same population. |
| Bjerre | 2019 | Previous efficacy study completed | FC Prostate Community was launched based upon positive findings in small-scale exploratory randomised control trial |  |
| Culos-Reed | 2018 | Scoping review + review of established programs + community consultation | Initial scoping review of existing programs, resources, and literature for lifestyle programs for prostate cancer. This included review of protocols for established programs and outreach to professionals operating existing program and industry-led workshops |  |
| Dolan | 2018 | Previous efficacy study completed | The Health, Exercise, Active Living, Therapeutic lifestyle (HEALTh) program evolved from a pilot study and uses the pre-existing cardiac rehabilitation framework for its structure |  |
| Kirkham | 2018 | Previous efficacy studies completed within same population + implementation barriers | Design considered 4 previous randomised control trials in same population and identified barriers and enablers to implementation |  |
| Kirkham | 2019 | Previous efficacy studies completed within same population + implementation barriers | Design considered previous randomised control trial and effectiveness results compared to efficacy study |  |
| Mackenzie | 2013 | Previous efficacy study completed | The Yoga Thrive program is a research-based, 7-week therapeutic yoga program that has been pilot-tested in both breast and prostate cancer patients |  |
| Rajotte | 2012 | Initial 12 months of program refinement | The first year of the program implementation focused on developing and refining training curriculum and program, determining effective and safe criteria for program eligibility, and training staff. Current study focuses on data collected during years 2 and 3 |  |
| Rogers | 2019 | Previous efficacy study completed | Efficacy established through previous trial, program is now being adapted for implementation and dissemination |  |
| Santa Mina | 2019 | Pilot phase before roll-out | From April 2014 to July 2015 a pilot phase of the program was run before ongoing roll-out |  |
| Swenson | 2014 | Program based on established model of care | Program based upon Cancer Survivorship Model of Care and training provided by Rocky Mountain Cancer Rehabilitation Institute |  |
| VanGerpen | 2013 | Previous literature + established model + program staff | Program development used an established cardiac rehabilitation model, coupled with literature review and program delivery staff |  |
| **Cost** | | | | |
| Beidas | 2014 | Intervention cost | Hybrid model of self-payment (US$416.50), co-payments (range US$0-$80 per session) or combination | Organisation – initial implementation cost approx. $US45, 000 (2018). The source of funding varies between donations, fundraising, research grants. Hybrid models of intervention funding including; non-for-profit donations, fee-for-service models, co-payment from participant |
| Bjerre | 2018 | Intervention cost | The cost of delivering the FG intervention was $US46,213 |  |
| Culos-Reed | 2018 | Intervention cost | Initial 12 weeks is no charge and then the maintenance phase applies a fee-for-service model |  |
| Culos-Reed | 2019 | Intervention Cost | Initial 12 weeks is no charge and then participants paid CA$99 per subsequent 12-week session |  |
| Dalzell | 2017 | Organisation cost | Organisation - private donations, targeted fundraising events and research grants |  |
| Haas | 2011 | Organisation cost + intervention cost | Organisation - initial start-up costs covered by a financial commitment from all oncology provider in the community. Additional support from Lance Armstrong Foundation and local businesspeople who provided treadmills at cost. Intervention – no cost to individuals |  |
| Heston | 2015 | Organisation cost + intervention cost | Organisation - supported by not-for-profit Intervention – no cost to individuals |  |
| Kirkham | 2016 | Intervention cost | Intervention cost $300 (approx.50% of participants receive scholarship to complete program) |  |
| Kirkham | 2018 | Organisation cost + intervention cost | Organisation - Annual costs include start-up $US3,055, personal $US40,906, program $US860, Total (1st year) $US44,821 (subsequent years) $US41,766 |  |
| Marker | 2018 | Intervention cost | $59/month to cover facility membership |  |
| Rogers | 2019 | Organisation cost | Variable based on location, however implementation cost reported to be $350 per person |  |
| Santa Mina | 2012 | Organisation cost + Intervention cost | Organisation - hospital foundation and fundraising initially covered cost. A cost-recovery model was implemented to cover ongoing expenses, which also includes donations from participants |  |
| **Feasibility** | | | | |
| Bjerre | 2018 | Adherence rate | 64% attendance at sessions (12 weeks)  59% attendance at sessions (6 months) | Attrition ranged between 56% - 22%. Total mean attrition rate was 38.8% (n=9) and 38.4% (n=7) for unique programs (patients discontinuing the program),  Attendance ranged between 83% - 30%. Total mean attendance rate was 61.6% (n=16) and 63.7% (n=15) of unique programs (number of attended sessions) |
| Brown | 2019 | Adherence rate + attrition rate | 80% mean attendance rate; 56% attrition rate |  |
| Cheifetz | 2014 | Attrition rate | 44% attrition rate |  |
| Cheifetz | 2015 | Attrition rate | 26% attrition rate (and 85.4% continued to exercise after completion of the program) |  |
| Culos-Reed | 2018 | Adherence rate | 40.3% attendance rate (average across class types) |  |
| Dolan | 2018 | Adherence rate | 66.6% attendance rate |  |
| Haas | 2011 | Attrition rate | 50% attrition rate, a phone-call was implemented to support participants engagement, and this decreased attrition rate to 30% |  |
| Haas | 2012 | Attrition rate | 40% attrition rate at 6 months |  |
| Heston | 2015 | Attendance rate | 79% attendance rate at sessions |  |
| Irwin | 2017 | Attendance rate | 83% attendance rate at sessions |  |
| Kimmel | 2014 | Attrition rate | Attrition rate is ~45% |  |
| Kirkham | 2016 | Attrition rate | 45% attrition rate |  |
| Kirkham | 2018 | Adherence rate + attrition rate | 22% attrition rate, 54% attendance rate (average across program phases) |  |
| Leach | 2015 | Attendance rate | 31% attendance rate at sessions |  |
| Leach | 2016 | Attendance rate | 62.5% attendance at sessions (12 weeks)  30% attendance at sessions (24 weeks) |  |
| Mackenzie | 2013 | Attendance rate | 72% attendance rate at sessions |  |
| Marker | 2018 | Attendance rate | 62% attendance rate at sessions |  |
| Noble | 2012 | Attrition rate | 31% attrition rate |  |
| Santa Mina | 2017 | Attendance rate | 56% attendance rate at sessions (average across total program) |  |
| Sherman | 2010 | Adherence rate | 82.5% attendance rate |  |
| Speed-Andrews | 2012 | Attendance rate | 63.9% attendance rate |  |
| **Fidelity** | | | | |
| Bjerre | 2018 | Likert scale | Football coach’s fidelity to the intervention manual was 3.8 (3.7–3.9), where 1 was non-adherence and 5 was perfect adherence. 8 hours of training |  |
| Brown | 2019 | Descriptive report of training | Program is delivered by Clinical Cancer Exercise Specialists certified by the University of Northern Colorado Cancer Rehabilitation Institute. They receive over 500 hours of training and patient contact and were certified by a written examination and practical evaluation. |  |
| Cheifetz | 2014 | Descriptive report of training | Staff received 12 hours of training and received ongoing, onsite mentoring by nurses and physiotherapists | Adherence to the intervention protocol was approx. 76% (n=1). Quality of program delivery was assured through staff training (n=5) |
| Culos-Reed | 2018 | Descriptive report of training | Quality of program delivery was complemented by cancer and exercise training, including online (21 hours) and in-person (8 hours) training |  |
| Heston | 2015 | Descriptive report of training | Staff undergo training to achieve certification as a LIVESTRONG® at the YMCA instructor (up to 47 hours) |  |
| Santa Mina | 2017 | Descriptive report of training | Quality of program delivery is complemented by staff undergoing compulsory training in exercise and cancer (8 hours) |  |
| **Penetration** | | | | |
| Kirkham | 2018 | Referral rate | Referral rate - 53% | Referral rate 53%(n=1) |
| **Sustainability** | | | | |
| Haas | 2012 | QOL - SF-36 + barriers and enablers | Significant improvement in physical (F=2.33; p=0.031) and mental (F= 3.36; p=0.003) components of SF-36 over time. Sustainability of program reported (in part) due to a phone call made by the Clinical Director to schedule the initial visit and train staff monitoring participants | Health outcome (QOL) was sustained over time, supported by clinical director role and trained staff (n=1) |
